# Preclinical Evidence That Mesoglycan Unfolds Complex Anti-Aging Effects in Photoaged Female Facial Skin

**DOI:** 10.3390/ijms26125787

**Published:** 2025-06-17

**Authors:** Assaf Zeltzer, Aviad Keren, Ralf Paus, Amos Gilhar

**Affiliations:** 1 Plastic & Reconstructive Surgery Department, Rambam Health Care Campus, Haifa 3525408, Israel; a_zeltzer@rambam.health.gov.il; 2 Skin Research Laboratory, Rappaport Faculty of Medicine, Technion—Israel Institute of Technology, Haifa 3200003, Israel; kaviad@technion.ac.il; 3 Dr. Phillip Frost Department of Dermatology & Cutaneous Surgery, University of Miami Miller School of Medicine, Miami, FL 33125, USA; rxp803@med.miami.edu; 4 CUTANEON—Skin & Hair Innovations, D-13125 Berlin, Germany

**Keywords:** senotherapeutics, skin aging, mesoglycan, angiogenesis, VEGF-A, skin rejuvenation, perfusion, inflammation, glycosaminoglycan, photoaging

## Abstract

Novel senotherapeutics are needed to reverse aging-related skin decline. The research question addressed was whether mesoglycan, a clinically approved glycosaminoglycan formulation known to enhance perfusion, angiogenesis, and VEGF-A signaling, possesses therapeutic potential for rejuvenating photo aged human skin. To test this, we treated full-thickness photoaged facial human skin samples (mean age: 72 ± 5 years) from seven women ex vivo. The samples were treated with topical or medium-delivered mesoglycan (100, 200, and 300 µM) for 6 days under serum-free conditions that accelerate skin aging. Biomarkers associated with aging were assessed using quantitative immunohistomorphometry. Mesoglycan treatment improved key skin aging biomarkers at all doses. Compared to vehicle-treated skin, mesoglycan broadly enhanced epidermal structure and function, improved pigmentation-related markers, reduced cellular senescence, boosted mitochondrial performance and antioxidant defenses, and improved dermal matrix structure and microvasculature density. Notably, mesoglycan also upregulated VEGF-A and VEGFR2, promoting skin rejuvenation. Medium-delivered mesoglycan produced stronger overall effects, while rete ridge reappearance was observed exclusively after topical application. Mesoglycan demonstrates senotherapeutic potential in photoaged human skin, acting via complementary pathways, including VEGF-A upregulation. Although medium-delivered mesoglycan yielded the greatest biomarker improvements topical application restored rete ridges, a sign of epidermal reorganization and also significantly enhanced basement membrane structure, pigmentation, mitochondrial function and antioxidant defenses, while avoiding systemic exposure, making it the safer and more feasible route for localized skin anti-aging.

## 1. Introduction

Photoaging is a primary contributor to the prominently visible aging phenotype of facial skin and accelerates the intrinsic skin aging process [1,2,3,4,5]. With life expectancy steadily increasing, the demand for safe and effective treatments that slow or, ideally, reverse cellular senescence, increased fragility, and both structural and functional degradation of photoaged skin [6,7,8,9] is expected to rise significantly [10]. However, despite the availability of a wide range of anti-aging treatments and products that claim efficacy, with the possible exception of retinoids [11,12], estradiol [13], melatonin [14], and possibly calcitriols [15], few have been demonstrated to effectively target key molecular markers of aging in mature human skin [11,12,13].

The demand for more effective anti-aging strategies arises not only from the growing desire to halt or reverse personal skin aging for psychological and sociocultural reasons but also because progressive skin senescence impairs normal skin functions, reduces wound healing capacity, increases the risk of skin cancer, and may be an indicator of several age-related morbidities [16]. Furthermore, chronic inflammatory skin diseases, glucocorticoid therapy, or chemotherapy can exacerbate aging processes in human skin [17]. Thus, identifying new candidate activities for anti-aging therapies is not only cosmetic, but of genuinely dermatological concern. The recent identification of vascular endothelial growth factor (VEGF-A) and VEGF receptor 2 (VEGFR2)-mediated signaling as a key driver of tissue rejuvenation in both mouse internal organs [18] and human skin [19] has paved the way for the discovery of new classes of potent anti-skin-aging agents. However, systemic VEGF-A administration carries the risk of serious adverse effects, including aberrant angiogenesis and the potential promotion of tumor growth [20]. Even topical application of this skin-rejuvenating growth factor [19] over large skin areas is technically very challenging, impractical, and not economically viable. Therefore, the field is challenged to come up with persuasive alternative anti-skin-aging strategies beyond photoprotection and optimized epidermal barrier management.

One attractive candidate senotherapeutic agent is mesoglycan, a composite of various gylcosaminoglycans (GAGs) derived from animal sources, namely heparan sulfate (HS), dermatan sulfate (DS), electrophoretically slow-moving heparin, and varying amounts of chondroitin sulfate (CS) [21,22,23]. Mesoglycan synergizes with VEGF [24,25,26] and has shown promise in wound healing management, e.g., by promoting keratinocyte migration and early differentiation [27,28,29,30,31,32,33] and supporting vascular function [25,34]. Reportedly, mesoglycan also mitigates microvascular dysfunction and oxidative stress and modulates inflammatory pathways [25,31] in a manner that might reduce “inflammaging” and slow cellular senescence [18,35,36]. Additionally, mesoglycan increased dermal expression of extracellular matrix (ECM)-related markers, such as collagen-associated staining and fibrillin-1, which are commonly diminished in aged skin and contribute to reduced firmness, elasticity, and structural support [14,37].

Therefore, this preclinical study aimed to evaluate the senotherapeutic efficacy of mesoglycan as a potential anti-skin-aging agent. Using a serum-free, standardized ex vivo human skin organ culture model that induces a markedly accelerated aging phenotype (“speed aging”) [38], we assessed how topically or medium-delivered mesoglycan impacts skin morphology and key biomarkers of intrinsic and extrinsic aging in aged female facial skin ex vivo. Mesoglycan is already marketed as an oral and topical treatment for chronic venous disease, hemorrhoid disease, and superficial vein thrombosis [25,26], which enhances its clinical relevance as a potential skin-senotherapeutic agent. Overall, our study provides proof of principle that the tested mesoglycan preparation exerts profound anti-aging activities in naturally photoaged human facial skin ex vivo.

## 2. Results

**Figure 1:**
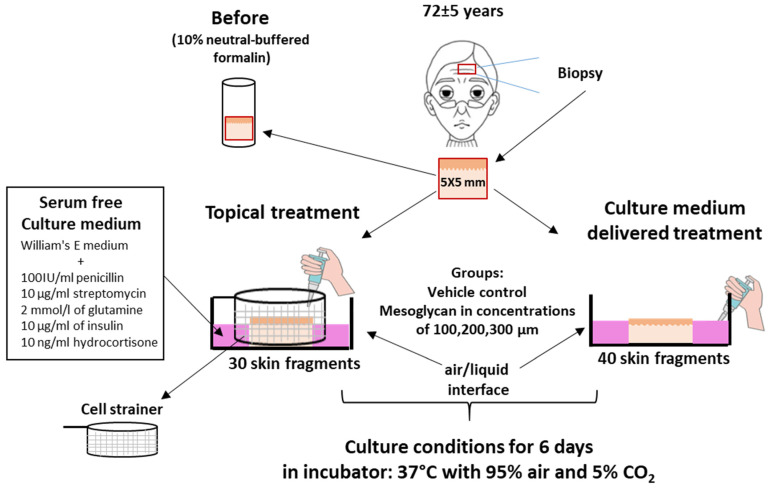
Ex vivo organ-culture setup for mesoglycan treatment of aged human facial skin. Full-thickness forehead skin fragments (5 × 5 mm; *n* = 70, ten per donor) were washed in William’s E medium supplemented with penicillin, streptomycin, glutamine, insulin, and hydrocortisone. For Topical treatment, 30 fragments were placed, epidermis side up, on cell strainers in six well plates containing 5.5 mL medium per well. For culture medium-delivered treatment, 40 fragments were floated epidermis-up in 24-well plates containing 1 mL supplemented medium per well. In both setups, explants were kept at the air/liquid interface and incubated for 6 days at 37 °C in 95% air/5% CO_2_, with medium changes every other day.

The impact of mesoglycan on biomarkers of aging in female photoaged facial skin ex vivo was assessed in serum-free full-thickness organ culture [14,19,38] as summarized in Figure 1, comparing the effects of topical with medium-delivered mesoglycan.

### 2.1. Rationale for Biomarker Selection

To comprehensively evaluate mesoglycan’s anti-aging effects, we selected a panel of biomarkers arranged anatomically from epidermis to dermis: in the epidermis, laminins were measured to assess basement-membrane integrity and epidermal–dermal cohesion [39,40,41], filaggrin to assess barrier function and hydration [42], collagen 17A1 for keratinocyte adhesion to the basement membrane [39,43,44,45], Ki-67 expression levels as a marker of basal-layer proliferative activity [46,47], p16^INK4A^ [14,19,48,49,50] and p-S6 as indicators of cellular senescence and mTORC1 signaling, respectively [14,51,52], Lamin B1 [3,19,53,54,55] and SIRT1 for nuclear integrity and longevity pathways [14,56,57]. As pigmentation readouts, Masson–Fontana histochemistry [58] alongside gp100 (PMEL) [59], MITF [60,61,62], and c-KIT [63] immunohistology were performed to evaluate melanin production and melanocyte function.

In the dermis, fibrillin-1 was assessed to elastic fiber content [14], and Masson’s trichrome histochemistry for overall collagen content and organization [64]. CD31 immunohistochemistry was used to quantify endothelial cells and microvascular density [65,66,67], and VEGF-A/VEGFR2 as drivers of angiogenesis and human skin rejuvenation [18,19,65,66,68,69,70].

Mitochondrial status was assessed by PGC1α [71,72,73], MTCO-1 and VDAC/porin [14,38,74,75,76]. Antioxidant defenses were probed by NRF-2 as the master regulator of oxidative-stress responses [57,77,78,79], HO-1 for heme degradation and cytoprotection [79,80], glutathione reductase for redox buffering [81], and PRDX (peroxiredoxin) for peroxide detoxification and cellular repair [80,82,83]. Together, this provides a comprehensive battery of skin aging-relevant read-outs.

### 2.2. Medium-Delivered Mesoglycan Improves Epidermal Morphology and Increases Thickness, Basal Layer Proliferation, and Skin Barrier Status in Photoaged Facial Skin

**Figure 2:**
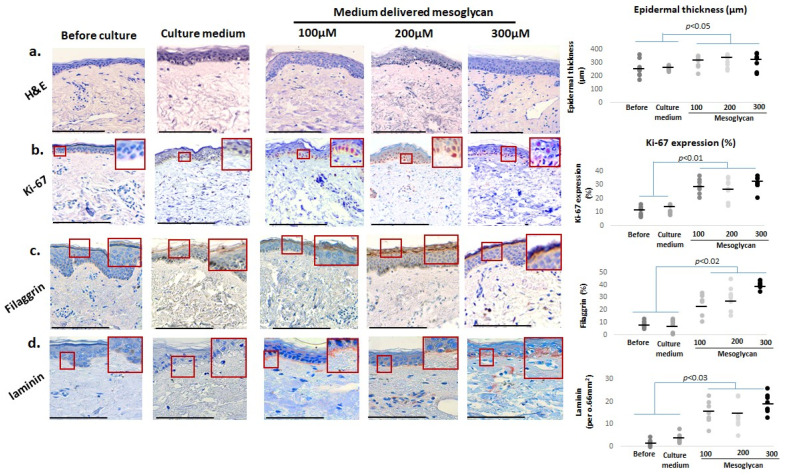
Medium-delivered mesoglycan enhances epidermal thickness, proliferation, and barrier function in photoaged facial skin ex vivo. Human skin was treated with medium-delivered mesoglycan (100–300 µM) under serum-free “speed-aging” conditions for 6 days. (**a**) Epidermal thickness significantly increased compared to baseline and medium-only controls, though rete ridges remained absent. (**b**) Expression of Ki-67, (**c**) filaggrin, and (**d**) laminin was significantly elevated, with filaggrin showing a dose-dependent effect. These changes were exclusive to mesoglycan-treated samples. Gray and black dots represent individual donors, and the horizontal bar indicates the group average. Red boxes on the micrographs mark the exact regions of interest that were quantified in the adjacent plots (see Section 4.7 for details). Data are mean ± SEM (*n* = 3 sections from 2 skin samples per group, 4 donors). Images were taken under ×200 magnification. Statistical significance: *p* < 0.05 (Shapiro–Wilk, One-way ANOVA, or Mann–Whitney U test). Scale bars: 50 µm.

First, we asked if medium-delivered mesoglycan improves key aging-associated epidermal parameters in photoaged female facial skin after addition to the organ culture medium. Quantitative histomorphometry (quantification was performed as described in Section 4.7) showed that mesoglycan significantly increased epidermal thickness in old photoaged facial skin compared to baseline and vehicle controls, even though rete ridges remained and did not show improved elongation (Figure 2a), a key morphological indicator of human epidermal rejuvenation [19].

Yet, mesoglycan treatment enhanced Ki-67 protein expression predominantly in basal layer keratinocytes, an indicator of proliferative activity (Figure 2b) [46,47]. Mesoglycan also significantly elevated filaggrin levels (Figure 2c) [37]. Moreover, laminin levels within the basement membrane were markedly elevated (Figure 2d) [39,40,41], suggesting improved dermo-epidermal-junction-associated features. Collectively, these results highlight that medium-delivered mesoglycan does indeed improve key epidermal parameters, including morphology, basal proliferation, and barrier-related markers, in photoaged female facial skin ex vivo.

### 2.3. Medium-Delivered Mesoglycan Enhances Pigment-Associated Markers in the Photoaged Human Epidermis Ex Vivo

While it has long been known that epidermal pigmentation declines during human skin aging and that its restoration is an indicator of skin rejuvenation [14,84], it has only recently become appreciated that senescent melanocytes can drive epidermal aging, e.g., by impairing basal keratinocyte proliferation and promoting their aging on multiple levels [85]. Therefore, we specifically interrogated the effects of medium-delivered mesoglycan on a battery of pigmentary read-outs. qIHC demonstrated that mesoglycan markedly improves the pigmentation of photoaged facial skin on several levels.

**Figure 3:**
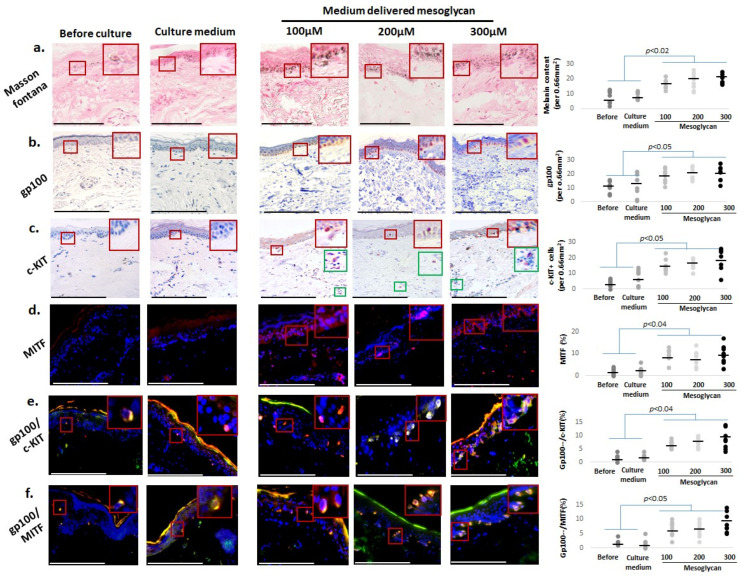
Medium-delivered mesoglycan enhances pigmentary markers in photoaged human epidermis ex vivo. Mesoglycan treatment significantly increased epidermal melanin content and expression of pigmentation-associated markers. (**a**) Fontana–Masson staining showed elevated melanin levels. Immunohistomorphometry confirmed upregulation of (**b**) gp100, (**c**) c-KIT, (**d**) MITF, and co-expression of (**e**) gp100/c-KIT and (**f**) gp100/MITF across all concentrations. Gray and black dots represent individual donors, and the horizontal bar indicates the group average. Red boxes on the micrographs mark the exact regions of interest that were quantified in the adjacent plots (see Section 4.7 for details). Data are mean ± SEM from three non-consecutive sections per group (two skin samples, four donors). Images were taken under ×200 magnification. Statistical analysis: Shapiro–Wilk, One-way ANOVA, or Mann–Whitney U test (*p* < 0.05). Scale bars: 50 µm.

Fontana–Masson histochemistry [58] revealed a significant increase in the epidermal melanin content in all mesoglycan-treated skin fragments, indicating enhanced melanogenesis (Figure 3a). Additionally, epidermal expression of the premelanosomal marker protein, gp100 [59], was significant increased compared to both the vehicle control and baseline levels, highlighting that mesoglycan activates melanocytes and promotes melanogenesis (Figure 3b). Intraepidermal immunoreactivity for the SCF receptor, c-KIT (CD117), and for MITF, the master transcription factor that governs melanogenesis and melanocyte biology [53,54,55,56,57,58,59,60], was also significantly increased compared to the vehicle control and baseline levels (Figure 3c,d), confirming melanocyte activation/reaction [60,63]. Moreover, double immunostaining for gp100 and c-KIT, as well as for gp100 and MITF, was also significantly enhanced when comparing these groups (Figure 3e,f).

Taken together, these findings demonstrate an impressive reactivation of the human epidermal pigmentary unit on multiple levels in old, photoaged facial epidermis ex vivo by medium-delivered mesoglycan, suggesting a skin rejuvenation effect [19] of the GAG composite preparation tested here.

### 2.4. Medium-Delivered Mesoglycan Improves Several Key Biomarkers of Skin Aging Ex Vivo

**Figure 4:**
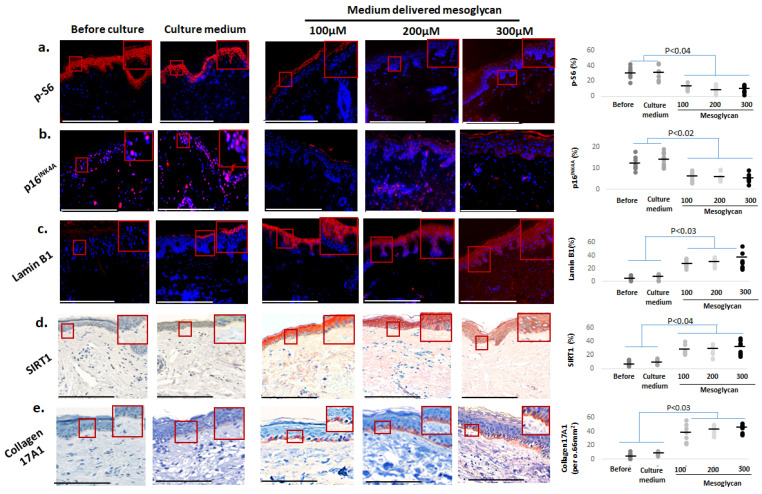
Medium-delivered mesoglycan improves key classical skin aging biomarkers in photoaged facial epidermis ex vivo. Medium-delivered administration of mesoglycan at 100 µM, 200 µm, and 300 µM reduces (**a**) p-S6 and (**b**) p16^INK4A^ while upregulating (**c**) Lamin B1 (**d**) SIRT1 and (**e**) Collagen17A1 compared to baseline and control medium culture. Quantitative immunohistomorphometry and representative images illustrate evaluated markers. Gray and black dots represent individual donors, and the horizontal bar indicates the group av-erage. Red boxes on the micrographs mark the exact regions of interest that were quantified in the adjacent plots (see Section 4.7 for details). Data are presented as mean ± SEM (*n* = 3 non-consecutive sections from 2 skin samples per group, taken from 4 donors). Images were taken under ×200 magnification. Statistical analysis (Shapiro–Wilk test, One-way ANOVA, or Mann–Whitney U test) considered *p* < 0.05 statistically significant. Scale bars: 50 µm.

A significant reduction in p-S6 [14,51,52] (Figure 4a) and p16^INK4A^ [14,19,48,49,50] (Figure 4b) was observed across all mesoglycan concentrations compared to the vehicle control and baseline levels. Moreover, mesoglycan treatment restored intraepidermal Lamin B1 levels [3,19,53,54,55,86] (Figure 4c) and significantly raised protein expression of SIRT1 [14,56,57] throughout the epidermis (Figure 4d). Furthermore, mesoglycan markedly increased collagen 17A1 expression in the epidermal basement membrane zone (Figure 4e) [39,43,44,45].

Taken together with the pigmentary effects reported above, the responses seen in these core biomarkers of skin aging and senescence strongly support that medium-delivered mesoglycan exerts profound ant-aging/senescence effects in photoaged facial skin ex vivo.

### 2.5. Mesoglycan Also Improves Mitochondrial Function Parameters and Cutaneous Oxidative Damage Defenses

**Figure 5:**
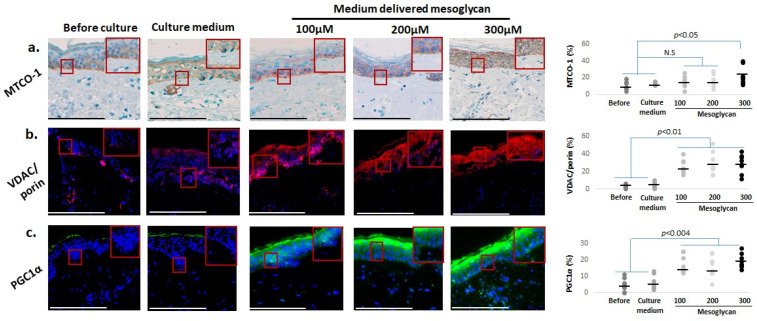
Medium-delivered mesoglycan enhances mitochondrial markers in aged human epidermis ex vivo. Treatment with mesoglycan (100–300 µM) significantly increased expression of mitochondrial proteins: (**a**) MTCO-1, with the highest levels at 300 µM; (**b**) VDAC/porin, involved in mitochondrial metabolite exchange; and (**c**) PGC1α, a regulator of mitochondrial biogenesis. Quantitative immunohistomorphometry and representative images illustrate these changes. Gray and black dots represent individual donors, and the horizontal bar indicates the group av-erage. Red boxes on the micrographs mark the exact regions of interest that were quantified in the adjacent plots (see Section 4.7 for details). Data: mean ± SEM from three non-consecutive sections per group (two skin samples, four donors). Images were taken under ×200 magnification. Statistical tests: Shapiro–Wilk, One-way ANOVA, or Mann–Whitney U (*p* < 0.05). Scale bars: 50 µm.

Given that skin aging is associated with progressive decline of mitochondrial function [57,87,88], we also assessed how mesoglycan impacted key mitochondrial read-outs. qIHC showed that, compared to baseline levels and the vehicle control, medium-delivered mesoglycan significantly increased intraepidermal protein expression of MTCO-1 [38] (Figure 5a). We had previously shown that MTCO-1 upregulation in human skin is correlated with increased mitochondrial activity [74,75], while reduced MTCO-1 immunoreactivity is associated with skin aging [38]. Epidermal protein levels for VDAC/porin protein [14,38,74,75,76] (Figure 5b), as well as those of PGC1α [70,71,72] (Figure 5c), were also significantly increased by medium-delivered mesoglycan.

**Figure 6:**
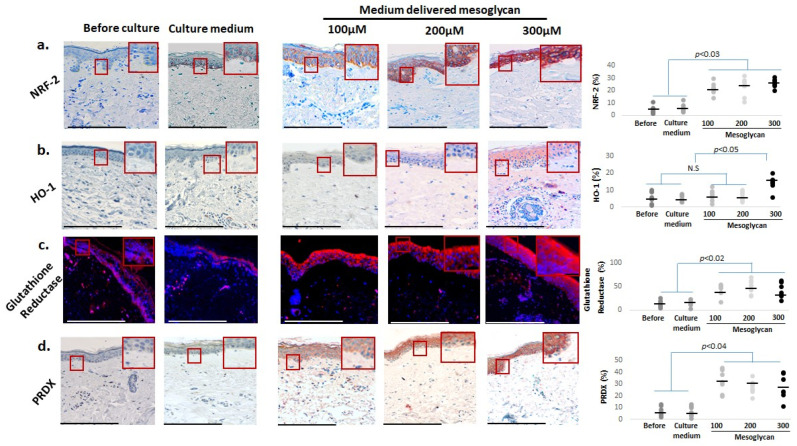
Medium-delivered mesoglycan enhances antioxidant markers in aged human epidermis ex vivo. Mesoglycan treatment (100–300 µM) significantly increased expression of key oxidative stress regulators: (**a**) NRF-2, (**b**) HO-1 (highest at 300 µM), (**c**) glutathione reductase, and (**d**) PRDX. Quantitative immunohistomorphometry and representative images demonstrate marker upregulation across treatment groups. Gray and black dots represent individual donors, and the horizontal bar indicates the group av-erage. Red boxes on the micrographs mark the exact regions of interest that were quantified in the adjacent plots (see Section 4.7 for details). Data are presented as mean ± SEM from three non-consecutive sections per group (two skin samples, four donors). Images were taken under ×200 magnification. Statistical analysis: Shapiro–Wilk, One-way ANOVA, or Mann–Whitney U test (*p* < 0.05). Scale bars: 50 µm.

Since mitochondria are the main sources of reactive oxygen species (ROS) production, we complemented these analyses with assessing key systems that mitigate oxidative damage. This showed that medium-delivered mesoglycan also increased the intraepidermal protein levels of NRF-2 [57,77,78,79] (Figure 6a) and of its downstream targets, HO-1 (Figure 6b) [79,80], glutathione reductase (Figure 6c) [81], and PRDX (Figure 6d) [80,82,83]. These findings suggest that mesoglycan can also counteract mitochondrial aging, as well as skin aging processes driven by the decline of mitochondrial function, and strengthens epidermal defense systems against oxidative damage in photoaged facial skin.

### 2.6. Mesoglycan Increases Dermal Collagen Staining and Fibrillin-1 Expression in Photoaged Human Skin Ex Vivo

**Figure 7:**
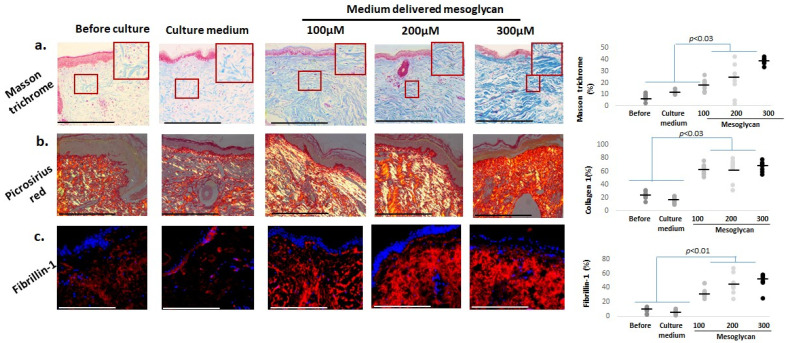
Medium-delivered mesoglycan enhances collagen and fibrillin-1 expression in aged human dermis ex vivo. Mesoglycan treatment (100–300 µM) significantly increased dermal staining intensity for (**a**) Masson’s trichrome staining with the strongest effect at 300 µM, and (**b**) Picrosirius red staining showed increased collagen deposition; (**c**) fibrillin-1 expression was also elevated, reflecting changes in elastic fiber-related structures. Quantitative immunohistomorphometry and representative images demonstrate these effects. Gray and black dots represent individual donors, and the horizontal bar indicates the group av-erage. Red boxes on the micrographs mark the exact regions of interest that were quantified in the adjacent plots (see Section 4.7 for details). Data: mean ± SEM from three non-consecutive sections per group (two skin samples, four donors). Images were taken under ×20 magnification. Statistics: Shapiro–Wilk, One-way ANOVA, or Mann–Whitney U test (*p* < 0.05). Scale bars: 50 µm.

Since collagen fragmentation, diminished biosynthesis, and elastic fiber damage are key contributors to dermal aging [89], we also assessed the dermal collagen by Masson’s trichrome staining [64] and Picrosirius red histochemistry [90]. Both were increased by mesoglycan (Figure 7a,b). Fibrillin-1 levels [14] were also significantly elevated by medium-delivered mesoglycan compared to baseline and vehicle control (Figure 7c), indicating substantial improvements in the extracellular matrix composition of photoaged dermis.

### 2.7. Medium-Delivered Mesoglycan Increases the Number of Endothelial Cells and VEGF-A/VEGFR2 Protein Expression in Photoaged Dermis

**Figure 8:**
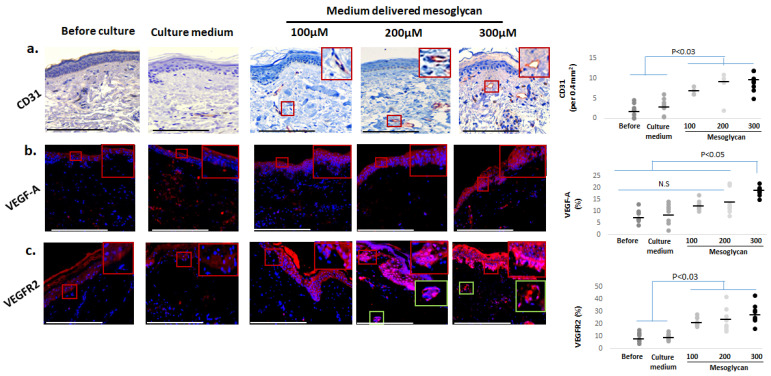
Medium-delivered mesoglycan increases vascularization markers in human skin ex vivo. Mesoglycan treatment significantly upregulated the following dermal vascular markers: (**a**) CD31+ cell numbers, indicating enhanced endothelial presence; (**b**) VEGF-A, elevated significantly at 300 µM; and (**c**) VEGFR2 expression. Quantitative immunohistomorphometry and representative images show consistent trends across treatment groups. Gray and black dots represent individual donors, and the horizontal bar indicates the group av-erage. Red and green boxes on the micrographs mark the exact regions of interest that were quantified in the adjacent plots (see Section 4.7 for details). Data are presented as mean ± SEM from three non-consecutive sections per group (two skin samples, four donors). Images were taken under ×200 magnification. Statistical analysis: Shapiro–Wilk, One-way ANOVA, or Mann–Whitney U test (*p* < 0.05). Scale bars: 50 µm.

Intrinsic and extrinsic aging of human skin is associated with reduced cutaneous microvasculature and reduced VEGFR1 and VEGF-A expression [18,19,65,66,67,69,70]. Therefore, it was interesting to note that, compared to controls, medium-delivered mesoglycan treatment significantly increased the number of CD31+ endothelial cells in the reticular and papillary dermis (Figure 8a). In addition, mesoglycan markedly elevated protein expression of VEGF-A—a key regulator of angiogenesis and recently implicated in human skin rejuvenation [18,19] (Figure 8b)—which was predominantly localized to the epidermis, particularly in the basal and suprabasal layers. VEGFR2 was also detected primarily in basal keratinocytes and in a limited number of dermal cells, including some with perivascular localization (Figure 8c).

### 2.8. Topical and Medium-Delivered Mesoglycan Elicit Differential Anti-Skin-Aging Effects Ex Vivo

**Figure 9:**
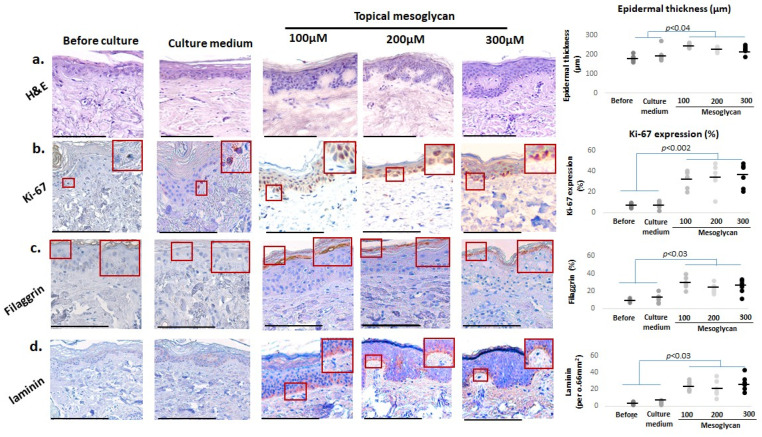
Topical mesoglycan enhances epidermal thickness, proliferation, and barrier markers in photoaged human skin ex vivo. Topical mesoglycan (100–300 µM) significantly increased (**a**) epidermal thickness with induction of rete ridges, (**b**) Ki-67 expression, (**c**) filaggrin levels, and (**d**) laminin expression. These effects were exclusive to mesoglycan-treated samples and absent in controls. Quantitative immunohistomorphometry supports these findings. Gray and black dots represent individual donors, and the horizontal bar indicates the group average. Red boxes on the micrographs mark the exact regions of interest that were quantified in the adjacent plots (see Section 4.7 for details). Data are shown as mean ± SEM from three non-consecutive sections per group (two skin samples, four donors). Images were taken under ×200 magnification. Statistical analysis: Shapiro–Wilk, One-way ANOVA, or Mann–Whitney U test (*p* < 0.05). Scale bars: 50 µm.

As shown in Supplementary Table S1, both topical (Figure 9 and Figures S1–S6) and medium-delivered mesoglycan treatments over 6 days significantly improved multiple skin aging biomarkers compared to baseline and the PEG4000 + PBS vehicle control. However, important differences were observed between the two treatment routes. Topical mesoglycan induced elongation of rete ridges (Figure 9a), a morphological feature often reduced in aged skin and indicative of a more complex epidermal–dermal interface. It also exerted stronger effects on markers involved in melanocyte function and pigmentation, including c-KIT (Supplementary Figure S1c) and MITF (Supplementary Figure S1d), as well as PGC1α (Supplementary Figure S3c), which supports mitochondrial biogenesis. Laminin (Figure 9d), important for basement membrane structure, also showed greater improvement following topical treatment. In contrast, medium-delivered mesoglycan produced a more pronounced increase in several key anti-aging and functional biomarkers. These included the barrier function marker filaggrin (Figure 9c); pigmentation-related proteins such as gp100 (Supplementary Figure S1b), gp100/c-KIT (Supplementary Figure S1e), and gp100/MITF ratios (Supplementary Figure S1f); the senescence-associated marker p16^INK4A^ (Supplementary Figure S2b); the longevity and anti-inflammatory marker SIRT1 (Supplementary Figure S2d); collagen 17A1, a matrix component linked to epithelial stem cell niches (Supplementary Figure S2e); the antioxidant enzyme PRDX (Supplementary Figure S4d); and the extracellular matrix component fibrillin-1 (Supplementary Figure S5c). Several biomarkers responded similarly to both topical and medium-delivered treatments, showing comparable levels of change across conditions. These included Ki-67 (Figure 9b) as an indicator of proliferative activity; p-S6 (Supplementary Figure S2a); Lamin B1 (Supplementary Figure S2c), associated with nuclear envelope stability and aging; MTCO-1 and VDAC/porin (Supplementary Figure S3a,b), related to mitochondrial function; NRF2, HO-1, and glutathione reductase (Supplementary Figure S4a–c), all markers of antioxidant defense; Masson–Fontana (Supplementary Figure S1a), Masson’s trichrome, and Picrosirius red staining (Supplementary Figure S5a,b) for collagen content; and CD31+ cell counts and VEGF-A/VEGFR2 expression (Supplementary Figure S6a–c), reflecting vascularization and angiogenic activity.

These findings underscore that topical and medium-delivered mesoglycan elicit partially overlapping yet distinct molecular and morphological anti-aging responses in human photoaged skin ex vivo. Understanding these differences is essential for guiding future clinical applications, especially when selecting the most appropriate route of administration for targeted therapeutic outcomes.

## 3. Discussion

Our ex vivo study, conducted using a highly sensitive “speed-aging” organ culture system [38], provides proof of principle that animal-derived glycosaminoglycans (GAGs), particularly mesoglycan, can exert substantial anti-aging effects in photoaged female facial skin. These effects were observed in both the epidermis and dermis using a broad range of classical skin aging-related protein-level biomarkers. The associated improvements in pigmentation, mitochondrial function, and oxidative damage repair suggest that mesoglycan has significant senotherapeutic potential. These findings highlight the need for clinical trials to explore mesoglycan’s long-term efficacy, especially for topical applications, potentially using PEG-containing vehicles to enhance skin penetration.

Efficient wound repair and intrinsic skin aging share overlapping molecular pathways; delayed healing—characterized by persistent senescent cells, impaired angiogenesis, and disordered extracellular-matrix turnover—not only underlies chronic non-healing wounds but also mirrors the molecular hallmarks of aged skin [3,91]. Conversely, interventions that promote pro-regenerative healing—by clearing senescent cells, restoring vascular networks, and normalizing matrix remodeling—simultaneously attenuate core features of cutaneous aging [92,93]. This duality underscores the mechanistic overlap between wound repair and skin rejuvenation and highlights why therapies targeting one process often benefit the other [88,94]. Recent work clarifies how each GAG in mesoglycan contributes to these multi-pathway benefits. Heparan-sulfate and heparin chains enhance VEGF-A and FGF-2 signaling in collagen–heparin scaffolds, greatly accelerating angiogenesis and dermal regeneration in a fetal-sheep wound mode [95]. In addition, heparan-sulfate/heparin chains unmask otherwise cryptic VEGF-binding motifs within fibronectin, thereby amplifying VEGF signaling and further stimulating neovascularization [96]. Dermatan sulfate incorporated into a bioactive three-layered skin substitute enhances FGF-10-driven keratinocyte migration and re-epithelialization [97], while chondroitin sulfate nano-formulations upregulate NRF-2/HO-1 and suppress NF-κB, providing antioxidant and anti-inflammatory “inflammaging” protection, as NRF-2 activity declines with age [98]. Finally, ex vivo application of GAG-enriched formulations significantly boosts dermal collagen deposition and skin hydration in aged human samples, underscoring translational relevance [99]. Together, these up-to-date findings provide a mechanistic framework linking mesoglycan’s components to the angiogenic, antioxidant, anti-inflammatory, and extracellular-matrix-rebuilding effects documented in our photo-aged skin model.

Notably, we observed differences in the anti-skin-aging effects following topical versus medium-delivered mesoglycan treatment ex vivo. For instance, rete ridge elongation, indicative of epidermal reorganization, was exclusively induced by topical application. Meanwhile, medium-delivered mesoglycan more significantly impacted markers related to barrier function, oxidative stress protection, and dermal matrix components like filaggrin, SIRT1, and collagen 17A1. These differences may reflect the limited penetration of high-molecular-weight GAG components, such as dermatan sulfate and heparan sulfate, across the epidermal barrier when applied topically. In contrast, lower-molecular-weight GAGs may preferentially accumulate in the epidermis or dermis depending on the delivery route. These route-dependent effects underline the need for future research to optimize delivery strategies and enhance the targeted therapeutic effects. Spatial proteomic analyses [100] may offer further insights into the localization and accumulation of mesoglycan components within specific skin compartments.

While mesoglycan has primarily been studied for its systemic effects in vascular, fibrotic, and metabolic disorders like chronic venous insufficiency, thrombophlebitis, and diabetic microangiopathy [25,34,101], our findings extend its therapeutic relevance to human skin aging. Previous studies have shown mesoglycan’s ability to enhance microcirculation, reduce endothelial dysfunction, and provide anti-inflammatory and cytoprotective effects [25]. In our study, we observed similar improvements in dermal vascular markers (CD31, VEGF-A, VEGFR2), mitochondrial function (PGC1α, MTCO-1, VDAC/porin), and antioxidant defenses (NRF-2, HO-1, PRDX). These findings suggest that mesoglycan acts through mechanisms common to both vascular and skin aging, supporting its potential as senotherapeutic agent in dermatology. Importantly, our study also addresses key gaps in the literature by demonstrating mesoglycan’s impact on skin architecture, pigmentation, and cellular senescence—areas that have been largely unexplored in the mesoglycan literature. Enhancements in melanocyte-associated markers such as gp100, MITF, and c-KIT indicate potential applications for treating hyperpigmentation and uneven skin tone, expanding mesoglycan’s potential beyond anti-aging to more cosmetic uses. Although improved pigmentation is very likely to reflect restoration of melanocyte function in aged epidermis [19,85,102,103] and will provide improved photoaging protection, future clinical trials will have to assess the possibility of cosmetically undesired hyperpigmentation.

Mesoglycan’s ability to suppress aging-associated markers like p16^INK4A^ and p-S6 while upregulating proteins such as Lamin B1, SIRT1, and collagen 17A1 positions it as a promising candidate in the growing class of senotherapeutic agents. These agents are capable of delaying or mitigating age-related cellular deterioration. While our findings are still limited and preliminary, they provide a valuable foundation for designing follow-up studies to validate mesoglycan’s efficacy in long-term in vivo models. Such studies should also optimize delivery strategies for improved skin penetration and evaluate its potential in combination with other skin senotherapeutics, e.g., [14,38]. One of the most intriguing findings in our study is the robust upregulation of VEGF-A and VEGFR2 protein expression under mesoglycan treatment. This is consistent with our previous work, where VEGF-A was identified as a key driver of human skin rejuvenation [19]. However, while VEGF-A upregulation is beneficial for rejuvenating skin, prolonged activation of this pathway can have adverse effects. For example, overexpression of VEGF-A promotes pathological angiogenesis that sustains tumor growth and facilitates metastatic spread. In fact, clinically, anti-VEGF therapeutics such as bevacizumab and small-molecule VEGFR inhibitors have become integral to cancer treatment by targeting this aberrant vasculature [104,105,106], and may be beneficial in psoriasis [107]. Elevated VEGF-A serum levels contribute to vascular leakage and inflammation in sepsis [106], exacerbate synovial angiogenesis in rheumatoid arthritis [108], andserve as a biomarker of disease activity and nephritis risk in systemic lupus erythematosus [109,110]. In the eye, chronic VEGF-A overexpression accelerates cataract formation [111], while in the skin, VEGF-A-driven angiogenesis underlies psoriatic plaque formation [112] and acute UV-induced sunburn [113].

As such, further research using VEGF-A neutralizing antibodies or VEGF/VEGFR antagonists will be necessary to clarify the functional role of VEGF-A/VEGFR signaling in mediating mesoglycan’s anti-aging effects and to assess its potential risks.

Despite the limitations of the organ culture model used in this study, which lacks innervation, functional vasculature, and physiological perfusion [114], the findings provide valuable insights into the potential of mesoglycan as a senotherapeutic agent. While the model does not replicate the full complexity of in vivo conditions, it serves as an instructive platform for understanding the mechanistic basis of mesoglycan’s effects on skin aging. However, caution is warranted when extrapolating these results to systemic applications. If skin aging is indeed a reliable indicator of systemic/organ aging [115,116,117], the anti-aging effects of mesoglycan observed in cultured skin may suggest potential benefits for other organs and even the brain. While no serious adverse effects have been reported in patients with venous thrombosis treated systemically with mesoglycan [118,119], chronic overstimulation of VEGF-A production could lead to unwanted angiogenesis and tumor growth [16,19,120]. These concerns emphasize the need for careful clinical monitoring when considering mesoglycan as a systemic senotherapeutic and suggest that topical applications, possibly in combination with microneedling, may offer a safer alternative for slowing or reversing human skin aging. In addition to the potential systemic effects, the range of biological effects associated with distinct GAGs, such as hyaluronic acid, chondroitin sulfate, dermatan sulfate, and heparan sulfate, in mammalian skin is vast [21,22,23]. However, the precise composition and concentration of individual GAGs in the mesoglycan preparation used in this study remain insufficiently characterized. Future research should focus on dissecting the mechanisms of action for each GAG component and understanding how they contribute to the anti-aging effects observed. This will provide a more robust framework for selecting the most effective mesoglycan components, particularly those that can raise intracutaneous VEGF-A levels and target key skin aging pathways, and will strengthen the mechanistic basis for future formulations [19].

In our previous work we have established VEGF-A as a pathway-specific reference compound for this same six-day “speed-aging” human-skin organ-culture model; in that study VEGF-A rapidly normalized basal-layer Ki-67, filaggrin, collagen I/III, fibrillin-1 and CD31, thereby validating the model’s responsiveness and providing a clear reference standard for epidermal, dermal, and vascular rejuvenation [19]. In fact, in this study, we had identified VEGF-A as the key driver of human skin rejuvenation ex vivo and in vivo. This serves as dependable reference data for comparing, wherever possible, the magnitude of mesoglycan’s anti-aging effects with those of VEGF-A across the relevant read-outs: filaggrin, collagen 17A1, Ki-67, p16^INK4A^, SIRT1, Masson–Fontana, c-KIT, CD31, VEGF-A, PGC1α, MTCO-1, NRF-2, HO-1, glutathione reductase, and PRDX (peroxiredoxin). Our pilot study results are limited by the fact that we had to utilize the very limited supply of valuable human facial skin (*n* = 7), for mesoglycan dose-finding studies and therefore could not possibly run an additional positive control arm like rhVEGF-A or, potentially, rhEGF for direct comparison with mesoglycan, and recommend to do so in a follow-up study.

While our pilot proof-of-concept study included a limited number of donors (*n* = 7), this sample size is relatively robust compared to other human skin organ culture studies, which often include 3–5 donors [19,74,121,122,123]. The scarcity of photoaged female human facial skin further limits donor availability. To mitigate inter-individual variability, we employed multiple technical replicates per donor and conducted paired within-donor comparisons. Rather than increasing the sample size for ex vivo experimentation, a more pertinent next step is to validate our findings in vivo, such as studying photoaged human skin xenotransplants on SCID/beige mice [19]. This approach would allow for a more comprehensive assessment of mesoglycan’s therapeutic potential in a physiologically relevant context. Importantly, topical mesoglycan, shown here to improve epidermal architecture, melanocyte function, basement membrane structure, mitochondrial function, and antioxidant defenses offer the safest, most practical approach for inducing localized skin rejuvenation, while short-term systemic mesoglycan administration may be best-suited to counter skin aging-associated vascular degeneration. Collectively, despite remaining mechanistic questions, these data underscore mesoglycan’s strong promise as a senotherapeutic for reversing key hallmarks of photoaged human facial skin.

## 4. Materials and Methods

### 4.1. Human Skin Sourcing

Aged facial skin samples were obtained from seven healthy female patients (mean age: 72 ± 5 years) undergoing routine plastic surgery (facelift). All human samples were obtained after informed written consent and ethics committee approved by the Rambam Health Care Campus Institutional Helsinki Committee (0182-14-RMB).

### 4.2. Skin Organ Culture

Full-thickness human facial skin samples were obtained from consenting donors. Two 5 × 5 mm skin fragments were excised per donor and either processed immediately or organ-cultured as described before [14,38]. The samples were divided into five groups:

Baseline Control (Pre-culture): Two fragments per donor were fixed in 10% neutral-buffered formalin for 24 h for subsequent analysis.

Two sets of experiments were performed:

1. Medium-delivered treatment of the test agent (*n* = 4 donors; two 5 × 5 mm skin fragments per donor): Mesoglycan was added directly to the culture medium at final concentrations of 100, 200, and 300 µM (Figure 2, Figure 3, Figure 4, Figure 5, Figure 6, Figure 7 and Figure 8).
2. Topical treatment (3 donors; two skin fragments per donor): Skin fragments were placed in a Cell Strainer (SPL, 93040). Mesoglycan was dissolved in PEG 4000 to increase viscosity and then applied to the skin surface via pipette, preventing diffusion into the underlying medium (Figure 9; Supplementary Figures S1–S6).

Vehicle Controls: Two fragments per donor were organ-cultured for 6 days in 1 mL of supplemented William’s E medium containing either PBS (used as the solvent for mesoglycan) or polyethylene glycol 4000 (PEG4000, 98%) plus 2% PBS, corresponding to the medium-delivered and topical treatment conditions, respectively, according to the assay design [14,38].

Mesoglycan (Prisma, Neopharmed Gentili): Two fragments per donor were cultured in serum-free, supplemented William’s E medium for 6 days with 100, 200, or 300 µM mesoglycan. PEG 4000 (98%), a common solvent and penetration enhancer, was used as the vehicle for topical mesoglycan application due to its ability to facilitate diffusion of large GAGs through the stratum corneum [124]. For medium-delivered treatment, mesoglycan was diluted in PBS, which also served as the vehicle. These tested mesoglycan doses were selected based on the previous demonstration that they promote angiogenesis and wound healing in mice [24]. For additional details, see Supplementary Information.

### 4.3. Histochemistry and Immunohistochemical and Immunofluorescence Staining

Hematoxylin and Eosin (H&E) staining was performed on paraffin sections as previously described [19]. Briefly, five-micrometer paraffin sections were deparaffinized and hydrated in distilled water. Following washing with tap water for 10 min, slides were incubated with Hematoxylin (Sigma-Aldrich Co. LLC, Wisconsin, WI, USA) for 10 min. Slides were than washed with tap water for 20 min and were placed in Eosin staining solution for 30 s. Following staining slides were dehydrated with ascending concentration of ethanol, cleared with xylene and mounted using DPX (Sigma-Aldrich Co. LLC).

For the immunohistochemical analysis, five-micrometer paraffin sections were used. Antigen retrieval was for 20 min at 90 °C in a microwave. Specimens were blocked for 30 min to prevent nonspecific binding and were incubated with 1ry Antibody (Ab) overnight, followed by the usual avidin biotin system, and by a wash and incubation with biotinylated 2nd Ab (Jackson ImmunoResearch, West Grove, PA, USA). The markers were revealed with AEC (red) substrate kit (Aminoethyl Carbazole Substrate kit, Vector laboratories, CA, USA). Sections were then mounted and analyzed under a light microscope.

For immunofluorescence analysis, the same protocol was used as above (immunohistochemical analysis) except slides were blocked for 1 h to prevent nonspecific binding and then incubated the primary antibodies overnight. The following day, slides were washed and incubated with secondary antibodies for 1 h. Slides were washed and incubated with DAPI for 10 min and then washed and mounted. Analysis was done by using the Confocal LSM 700 Upright Microscope (Zeiss, Oberkochen, Germany).

For VEGF-A, VEGFR2, Fibrillin-1, Masson trichrome, NRF-2, MTCO-1, PGC1α, Lamin B1, p-S6, VDAC/porin, Picrosirius red, HO-1, glutathione reductase, PRDX, filaggrin, and SIRT1, image analysis was performed using ImageJ software (Version 1.54j). Staining was calculated as the percentage of staining coverage. For Ki-67, p16^INK4A^, laminin, c-KIT, gp100, and collagen 17A1, the positive cells in the dermal compartment were counted in an area of 0.66 mm^2^. CD31-positive blood vessels were counted within an area of 0.4 mm^2^.

Protein immunoreactivity was evaluated using qIHM on specifically designated reference areas within non-consecutive sections as previously described [14,19,38,74,125], analyzing three sections per condition across two distinct skin fragments for each biomarker per donor, sourced from a total of 7 independent organ cultures. (For technical details see Supplementary Material and Methods and Tables S1 and S2).

### 4.4. Masson–Fontana Staining

(Abcam) was performed as described by us [126]. Briefly, five-micrometer paraffin sections were deparaffinized and hydrated in distilled water. Slides were placed in mixed ammoniacal silver solution in a 58–60 °C water bath and allowed adequate time for the temperature to equilibrate. Slides were then placed in warmed ammoniacal silver solution for 30–60 min or until the tissue section became yellowish/brown in color. Counterstaining was performed with Nuclear Fast Red Solution for 5 min.

### 4.5. Picrosirius Red Staining

(Abcam) was performed as described by us [19]. Five-micrometer paraffin sections were deparaffinized and hydrated in distilled water. Slides were covered in Picrosirius red solution and were incubated for 60 min at room temperature. Slides were then placed in acetic acid solution and immidiatley washed with absolute alcohol. Slides were then dehydrated, cleared with xylen, and mounted using DPX (Sigma-Aldrich Co. LLC).

### 4.6. Determination of Epidermal Thickness

Histological assessment of the skin was performed using light microscopy. Epidermal thickness was determined using an ocular micrometer at a minimum of 50 points along the epidermis selected to represent points of maximal and minimal thickness. The thickness of the suprapapillary plate was measured similarly at 50 points for each sample.

### 4.7. Quantification of Marker Staining

**Epidermal Markers**

Filaggrin: Measured as % intensity within the stratum corneum.

Cell-counted markers: Ki-67, p16^INK4A^, MITF, gp100/c-KIT, gp100/MITF

Quantified by counting the % of positive basal-layer keratinocytes (lowest two cell layers; 0.66 mm^2^ field).

Basal-layer histochemistry: Masson–Fontana, gp100, c-KIT, laminin, collagen 17A1. Counted as positive cells per 0.66 mm^2^ basal-layer field.

Fluorescence-intensity markers: NRF-2, HO-1, glutathione reductase, PRDX, SIRT-1, PGC1α, MTCO-1, p-S6, VDAC/porin, Lamin B1, VEGF-A, VEGFR2. Measured by mean fluorescence intensity over the full epidermal thickness (excluding stratum corneum).

**Dermal Markers**

Matrix staining: Masson’s trichrome, Picrosirius red, Fibrillin-1. Mean staining intensity in the upper 150 μm of the subepidermal dermis (papillary dermis).

Endothelial cells: CD31. Counted as positive vessels per 0.4 mm^2^ within the first 200 μm of subepidermal dermis.

**Imaging Details:**

Histochemistry and Immunohistochemical Staining: captured on an AxioLab 5 (Zeiss, Oberkochen, Germany) bright-field microscope.

Immunofluorescence: acquired on a Zeiss LSM 700 confocal at 200×, with identical exposure settings for each marker across all donors.

Analysis: Quantitative immunohistomorphometry (qIHM) performed in ImageJ (NIH).

### 4.8. Statistical Analysis

Data are presented as the mean ± standard deviation (SD), and *p* values of <0.05 were regarded as significant.

Gaussian distribution of the data was analyzed using the Shapiro–Wilk test. Significant differences were analyzed using the Mann–Whitney test (comparison between one set of data and before transplantation and no treatment) or One-way ANOVA (comparison between multiple sets of data).

## Data Availability

The datasets used and/or analyzed during the current study are available from the corresponding authors on reasonable request.

## Supplementary Materials

The following supporting information can be downloaded at: https://www.mdpi.com/article/10.3390/ijms26125787/s1. References [127,128,129] are cited in the supplementary materials.

## References

[1] Ho C.Y., Dreesen O. (2021). Faces of cellular senescence in skin aging. Mech. Ageing Dev..

[2] Langton A.K., Watson R.E.B. (2021). Identification of novel skin ageing genes: Evidence from across the pigmentary continuum. Br. J. Dermatol..

[3] Chin T., Lee X.E., Ng P.Y., Lee Y., Dreesen O. (2023). The role of cellular senescence in skin aging and age-related skin pathologies. Front. Physiol..

[4] Yu G.T., Ganier C., Allison D.B., Tchkonia T., Khosla S., Kirkland J.L., Lynch M.D., Wyles S.P. (2025). Mapping epidermal and dermal cellular senescence in human skin aging. Aging Cell.

[5] Liu Z., Liang Q., Ren Y., Guo C., Ge X., Wang L., Cheng Q., Luo P., Zhang Y., Han X. (2023). Immunosenescence: Molecular mechanisms and diseases. Signal Transduct. Target. Ther..

[6] Griffiths T.W., Watson R.E.B., Langton A.K. (2023). Skin ageing and topical rejuvenation strategies. Br. J. Dermatol..

[7] López-Otín C., Blasco M.A., Partridge L., Serrano M., Kroemer G. (2023). Hallmarks of aging: An expanding universe. Cell.

[8] Csekes E., Račková L. (2021). Skin Aging, Cellular Senescence and Natural Polyphenols. Int. J. Mol. Sci..

[9] Kohl E., Steinbauer J., Landthaler M., Szeimies R.M. (2011). Skin ageing. J. Eur. Acad. Dermatol. Venereol..

[10] Sadick N., Pannu S., Abidi Z., Arruda S. (2023). Topical Treatments for Photoaged Skin. J. Drugs Dermatol..

[11] Lau M., Mineroff Gollogly J., Wang J.Y., Jagdeo J. (2024). Cosmeceuticals for antiaging: A systematic review of safety and efficacy. Arch. Dermatol. Res..

[12] Halai P., Kiss O., Wang R., Chien A.L., Kang S., O’Connor C., Bell M., Griffiths C.E.M., Watson R.E.B., Langton A.K. (2024). Retinoids in the treatment of photoageing: A histological study of topical retinoid efficacy in black skin. J. Eur. Acad. Dermatol. Venereol..

[13] Mellody K.T., Kendall A.C., Wray J.R., Foster A.R., Langton A.K., Costello P., Newton V.L., Bell M., Griffiths C.E.M., Nicolaou A. (2022). Influence of menopause and hormone replacement therapy on epidermal ageing and skin biomechanical function. J. Eur. Acad. Dermatol. Venereol..

[14] Samra T., Gomez-Gomez T., Linowiecka K., Akhundlu A., Lopez de Mendoza G., Gompels M., Lee W.W., Gherardini J., Chéret J., Paus R. (2023). Melatonin Exerts Prominent, Differential Epidermal and Dermal Anti-Aging Properties in Aged Human Eyelid Skin Ex Vivo. Int. J. Mol. Sci..

[15] Bhattarai H.K., Shrestha S., Rokka K., Shakya R. (2020). Vitamin D, Calcium, Parathyroid Hormone, and Sex Steroids in Bone Health and Effects of Aging. J. Osteoporos..

[16] Mekić S., Pardo L.M., Gunn D.A., Jacobs L.C., Hamer M.A., Ikram M.A., Vinke E.J., Vernooij M.W., Haarman A.E.G., Thee E.F. (2023). Younger facial looks are associate with a lower likelihood of several age-related morbidities in the middle-aged to elderly. Br. J. Dermatol..

[17] Baechle J.J., Chen N., Makhijani P., Winer S., Furman D., Winer D.A. (2023). Chronic inflammation and the hallmarks of aging. Mol. Metab..

[18] Grunewald M., Kumar S., Sharife H., Volinsky E., Gileles-Hillel A., Licht T., Permyakova A., Hinden L., Azar S., Friedmann Y. (2021). Counteracting age-related VEGF signaling insufficiency promotes healthy aging and extends life span. Science.

[19] Keren A., Bertolini M., Keren Y., Ullmann Y., Paus R., Gilhar A. (2022). Human organ rejuvenation by VEGF-A: Lessons from the skin. Sci. Adv..

[20] Zhan H., Li H., Liu C., Cheng L., Yan S., Li Y. (2021). Association of Circulating Vascular Endothelial Growth Factor Levels With Autoimmune Diseases: A Systematic Review and Meta-Analysis. Front. Immunol..

[21] Sammon D., Krueger A., Busse-Wicher M., Morgan R.M., Haslam S.M., Schumann B., Briggs D.C., Hohenester E. (2023). Molecular mechanism of decision-making in glycosaminoglycan biosynthesis. Nat. Commun..

[22] Ricard-Blum S., Vivès R.R., Schaefer L., Götte M., Merline R., Passi A., Heldin P., Magalhães A., Reis C.A., Skandalis S.S. (2024). A biological guide to glycosaminoglycans: Current perspectives and pending questions. FEBS J..

[23] Shi D., Sheng A., Chi L. (2021). Glycosaminoglycan-Protein Interactions and Their Roles in Human Disease. Front. Mol. Biosci..

[24] Belvedere R., Novizio N., Morello S., Petrella A. (2022). The combination of mesoglycan and VEGF promotes skin wound repair by enhancing the activation of endothelial cells and fibroblasts and their cross-talk. Sci. Rep..

[25] Gallo G., Picciariello A., Tufano A., Camporese G. (2024). Clinical evidence and rationale of mesoglycan to treat chronic venous disease and hemorrhoidal disease: A narrative review. Updates Surg..

[26] Camporese G., Bernardi E., Bortoluzzi C., Noventa F., Simioni P., METRO Investigator Study Group (2024). Mesoglycan for the secondary prevention of superficial vein thrombosis: A randomized, controlled, double-blind study (METRO Study)-rationale and protocol. J. Thromb. Thrombolysis.

[27] Mottola S., Viscusi G., Belvedere R., Petrella A., De Marco I., Gorrasi G. (2024). Production of mono and bilayer devices for wound dressing by coupling of electrospinning and supercritical impregnation techniques. Int. J. Pharm..

[28] Zhou Y., Tian Y., Zhang M. (2024). Technical development and application of supercritical CO_2_ foaming technology in PCL foam production. Sci. Rep..

[29] Bizzarro V., Belvedere R., Pessolano E., Parente L., Petrella F., Perretti M., Petrella A. (2019). Mesoglycan induces keratinocyte activation by triggering syndecan-4 pathway and the formation of the annexin A1/S100A11 complex. J. Cell Physiol..

[30] Pessolano E., Belvedere R., Bizzarro V., Franco P., Marco I., Petrella F., Porta A., Tosco A., Parente L., Perretti M. (2019). Annexin A1 Contained in Extracellular Vesicles Promotes the Activation of Keratinocytes by Mesoglycan Effects: An Autocrine Loop Through FPRs. Cells.

[31] Belvedere R., Bizzarro V., Parente L., Petrella F., Petrella A. (2017). The Pharmaceutical Device Prisma^®^ Skin Promotes in Vitro Angiogenesis through Endothelial to Mesenchymal Transition during Skin Wound Healing. Int. J. Mol. Sci..

[32] Belvedere R., Bizzarro V., Parente L., Petrella F., Petrella A. (2018). Effects of Prisma^®^ Skin dermal regeneration device containing glycosaminoglycans on human keratinocytes and fibroblasts. Cell Adhes. Migr..

[33] Mottola S., Viscusi G., Iannone G., Belvedere R., Petrella A., De Marco I., Gorrasi G. (2023). Supercritical Impregnation of Mesoglycan and Lactoferrin on Polyurethane Electrospun Fibers for Wound Healing Applications. Int. J. Mol. Sci..

[34] Valvano A., Bosso G., Apuzzi V., Riccone F., Saccà L., Oliviero U. (2015). Mesoglycan improves vascular reactivity and insulin sensitivity in patients with metabolic syndrome. Atherosclerosis.

[35] Zhang J., Xia B., Wakefield J.S., Elias P.M., Wang X. (2025). The Role and Implications of Epidermal Dysfunction in the Pathogenesis of Inflammaging. J. Investig. Dermatol..

[36] Singh A., Schurman S.H., Bektas A., Kaileh M., Roy R., Wilson D.M., Sen R., Ferrucci L. (2024). Aging and Inflammation. Cold Spring Harb. Perspect. Med..

[37] Pessolano E., Belvedere R., Novizio N., Filippelli A., Perretti M., Whiteford J., Petrella A. (2021). Mesoglycan connects Syndecan-4 and VEGFR2 through Annexin A1 and formyl peptide receptors to promote angiogenesis in vitro. FEBS J..

[38] van Lessen M., Mardaryev A., Broadley D., Bertolini M., Edelkamp J., Kückelhaus M., Funk W., Bíró T., Paus R. (2024). ‘Speed-ageing’ of human skin in serum-free organ culture ex vivo: An instructive novel assay for preclinical human skin ageing research demonstrates senolytic effects of caffeine and 2,5-dimethylpyrazine. Exp. Dermatol..

[39] Jeong S., Yoon S., Kim S., Jung J., Kor M., Shin K., Lim C., Han H.S., Lee H., Park K.Y. (2019). Anti-Wrinkle Benefits of Peptides Complex Stimulating Skin Basement Membrane Proteins Expression. Int. J. Mol. Sci..

[40] Iriyama S., Yasuda M., Nishikawa S., Takai E., Hosoi J., Amano S. (2020). Decrease of laminin-511 in the basement membrane due to photoaging reduces epidermal stem/progenitor cells. Sci. Rep..

[41] Byun K.A., Oh S., Batsukh S., Kim M.J., Lee J.H., Park H.J., Chung M.S., Son K.H., Byun K. (2023). The Extracellular Matrix Vitalizer RATM Increased Skin Elasticity by Modulating Mitochondrial Function in Aged Animal Skin. Antioxidants.

[42] Li L., Liu Y., Chang R., Ye T., Li Z., Huang R., Wang Z., Deng J., Xia H., Yang Y. (2024). Dermal Injection of Recombinant Filaggrin-2 Ameliorates UVB-Induced Epidermal Barrier Dysfunction and Photoaging. Antioxidants.

[43] Liu N., Matsumura H., Kato T., Ichinose S., Takada A., Namiki T., Asakawa K., Morinaga H., Mohri Y., De Arcangelis A. (2019). Stem cell competition orchestrates skin homeostasis and ageing. Nature.

[44] Liu Y., Ho C., Wen D., Sun J., Huang L., Gao Y., Li Q., Zhang Y. (2022). Targeting the stem cell niche: Role of collagen XVII in skin aging and wound repair. Theranostics.

[45] Nanba D., Toki F., Asakawa K., Matsumura H., Shiraishi K., Sayama K., Matsuzaki K., Toki H., Nishimura E.K. (2021). EGFR-mediated epidermal stem cell motility drives skin regeneration through COL17A1 proteolysis. J. Cell Biol..

[46] Endl E., Hollmann C., Gerdes J. (2001). Antibodies against the Ki-67 protein: Assessment of the growth fraction and tools for cell cycle analysis. Methods Cell Biol..

[47] Schlüter C., Duchrow M., Wohlenberg C., Becker M.H., Key G., Flad H.D., Gerdes J. (1993). The cell proliferation-associated antigen of antibody Ki-67: A very large, ubiquitous nuclear protein with numerous repeated elements, representing a new kind of cell cycle-maintaining proteins. J. Cell Biol..

[48] Zhao H., Liu Z., Chen H., Han M., Zhang M., Liu K., Jin H., Liu X., Shi M., Pu W. (2024). Identifying specific functional roles for senescence across cell types. Cell.

[49] Subramanian P., Sayegh S., Laphanuwat P., Devine O.P., Fantecelle C.H., Sikora J., Chambers E.S., Karagiannis S.N., Gomes D.C.O., Kulkarni A. (2024). Multiple outcomes of the germline p16^INK4a^ mutation affecting senescence and immunity in human skin. Aging Cell.

[50] Lyons C.E., Pallais J.P., McGonigle S., Mansk R.P., Collinge C.W., Yousefzadeh M.J., Baker D.J., Schrank P.R., Williams J.W., Niedernhofer L.J. (2025). Chronic social stress induces p16-mediated senescent cell accumulation in mice. Nat. Aging.

[51] Suzuki T., Chéret J., Scala F.D., Akhundlu A., Gherardini J., Demetrius D.L., O’Sullivan J.D.B., Kuka Epstein G., Bauman A.J., Demetriades C. (2023). mTORC1 activity negatively regulates human hair follicle growth and pigmentation. EMBO Rep..

[52] Gallage S., Irvine E.E., Barragan Avila J.E., Reen V., Pedroni S.M.A., Duran I., Ranvir V., Khadayate S., Pombo J., Brookes S. (2024). Ribosomal S6 kinase 1 regulates inflammaging via the senescence secretome. Nat. Aging.

[53] Matias I., Diniz L.P., Damico I.V., Araujo A.P.B., Neves L.D.S., Vargas G., Leite R.E.P., Suemoto C.K., Nitrini R., Jacob-Filho W. (2022). Loss of lamin-B1 and defective nuclear morphology are hallmarks of astrocyte senescence in vitro and in the aging human hippocampus. Aging Cell.

[54] En A., Takemoto K., Yamakami Y., Nakabayashi K., Fujii M. (2024). Upregulated expression of lamin B receptor increases cell proliferation and suppresses genomic instability: Implications for cellular immortalization. FEBS J..

[55] Lv T., Wang C., Zhou J., Feng X., Zhang L., Fan Z. (2024). Mechanism and role of nuclear laminin B1 in cell senescence and malignant tumors. Cell Death Discov..

[56] Xu C., Wang L., Fozouni P., Evjen G., Chandra V., Jiang J., Lu C., Nicastri M., Bretz C., Winkler J.D. (2020). SIRT1 is downregulated by autophagy in senescence and ageing. Nat. Cell Biol..

[57] Zhang C., Gao X., Li M., Yu X., Huang F., Wang Y., Yan Y., Zhang H., Shi Y., He X. (2023). The role of mitochondrial quality surveillance in skin aging: Focus on mitochondrial dynamics, biogenesis and mitophagy. Ageing Res. Rev..

[58] Cui X., Mi T., Zhang H., Gao P., Xiao X., Lee J., Guelakis M., Gu X. (2024). Glutathione amino acid precursors protect skin from UVB-induced damage and improve skin tone. J. Eur. Acad. Dermatol. Venereol..

[59] Sevilla A., Chéret J., Lee W., Paus R. (2023). Concentration-dependent stimulation of melanin production as well as melanocyte and keratinocyte proliferation by melatonin in human eyelid epidermis. Exp. Dermatol..

[60] Park Y.J., Kim J.C., Kim Y., Kim Y.H., Park S.S., Muther C., Tessier A., Lee G., Gendronneau G., Forestier S. (2023). Senescent melanocytes driven by glycolytic changes are characterized by melanosome transport dysfunction. Theranostics.

[61] Gelmi M.C., Houtzagers L.E., Strub T., Krossa I., Jager M.J. (2022). MITF in Normal Melanocytes, Cutaneous and Uveal Melanoma: A Delicate Balance. Int. J. Mol. Sci..

[62] Cheli Y., Ohanna M., Ballotti R., Bertolotto C. (2010). Fifteen-year quest for microphthalmia-associated transcription factor target genes. Pigment. Cell Melanoma Res..

[63] Kapur R., Everett E.T., Uffman J., McAndrews-Hill M., Cooper R., Ryder J., Vik T., Williams D.A. (1997). Overexpression of human stem cell factor impairs melanocyte, mast cell, and thymocyte development: A role for receptor tyrosine kinase-mediated mitogen activated protein kinase activation in cell differentiation. Blood.

[64] Cui B., Wang Y., Jin J., Yang Z., Guo R., Li X., Yang L., Li Z. (2022). Resveratrol Treats UVB-Induced Photoaging by Anti-MMP Expression, through Anti-Inflammatory, Antioxidant, and Antiapoptotic Properties, and Treats Photoaging by Upregulating VEGF-B Expression. Oxid. Med. Cell Longev..

[65] Liu Y.H., Brunner L.M., Rebling J., Ben-Yehuda Greenwald M., Werner S., Detmar M., Razansky D. (2022). Non-invasive longitudinal imaging of VEGF-induced microvascular alterations in skin wounds. Theranostics.

[66] Xiang T., Sun F., Liu T., Zhao J., Yang J., Ouyang D., Chen H., Zhu Q., Wang Q., Li Y. (2024). EBV-associated epithelial cancers cells promote vasculogenic mimicry formation via a secretory cross-talk with the immune microenvironment. Theranostics.

[67] Zhou S., Li Z., Li X., Ye Y., Wang M., Jiang J., Tao L., Wang Y., Tung C.-T., Chung Y. (2024). Crosstalk between endothelial cells and dermal papilla entails hair regeneration and angiogenesis during aging. J. Adv. Res..

[68] Baffert F., Thurston G., Rochon-Duck M., Le T., Brekken R., McDonald D.M. (2004). Age-related changes in vascular endothelial growth factor dependency and angiopoietin-1-induced plasticity of adult blood vessels. Circ. Res..

[69] Pérez-Gutiérrez L., Ferrara N. (2023). Biology and therapeutic targeting of vascular endothelial growth factor A. Nat. Rev. Mol. Cell Biol..

[70] Wang L., Liu W.Q., Broussy S., Fang H. (2024). Recent advances of anti-angiogenic inhibitors targeting VEGF/VEGFR axis. Front. Pharmacol..

[71] Rius-Pérez S., Torres-Cuevas I., Millán I., Ortega Á.L., Pérez S. (2020). PGC-1α, Inflammation, and Oxidative Stress: An Integrative View in Metabolism. Oxidative Med. Cell. Longev..

[72] Souder D.C., McGregor E.R., Clark J.P., Rhoads T.W., Porter T.J., Eliceiri K.W., Moore D.L., Puglielli L., Anderson R.M. (2025). Neuron-specific isoform of PGC-1α regulates neuronal metabolism and brain aging. Nat. Commun..

[73] Luo Y., Bollag W.B. (2024). The Role of PGC-1α in Aging Skin Barrier Function. Cells.

[74] Vidali S., Chéret J., Giesen M., Haeger S., Alam M., Watson R.E.B., Langton A.K., Klinger M., Knuever J., Funk W. (2016). Thyroid Hormones Enhance Mitochondrial Function in Human Epidermis. J. Investig. Dermatol..

[75] Vidali S., Knuever J., Lerchner J., Giesen M., Bíró T., Klinger M., Kofler B., Funk W., Poeggeler B., Paus R. (2014). Hypothalamic-pituitary-thyroid axis hormones stimulate mitochondrial function and biogenesis in human hair follicles. J. Investig. Dermatol..

[76] Ham S.J., Lee D., Yoo H., Jun K., Shin H., Chung J. (2020). Decision between mitophagy and apoptosis by Parkin via VDAC1 ubiquitination. Proc. Natl. Acad. Sci. USA.

[77] Haslam I.S., Jadkauskaite L., Szabó I.L., Staege S., Hesebeck-Brinckmann J., Jenkins G., Bhogal R.K., Lim F.L., Farjo N., Farjo B. (2017). Oxidative Damage Control in a Human (Mini-) Organ: Nrf2 Activation Protects against Oxidative Stress-Induced Hair Growth Inhibition. J. Investig. Dermatol..

[78] Kahremany S., Hofmann L., Gruzman A., Dinkova-Kostova A.T., Cohen G. (2022). NRF2 in dermatological disorders: Pharmacological activation for protection against cutaneous photodamage and photodermatosis. Free Radic. Biol. Med..

[79] O’Rourke S.A., Shanley L.C., Dunne A. (2024). The Nrf2-HO-1 system and inflammaging. Front. Immunol..

[80] Forman H.J., Zhang H. (2021). Targeting oxidative stress in disease: Promise and limitations of antioxidant therapy. Nat. Rev. Drug Discov..

[81] Kanamori A., Egawa N., Yamasaki S., Ikeda T., da Rocha M.J., Bortolatto C.F., Savegnago L., Brüning C.A., Iwaoka M. (2024). Antioxidative and Antiglycative Stress Activities of Selenoglutathione Diselenide. Pharmaceuticals.

[82] Wu M., Deng C., Lo T.H., Chan K.Y., Li X., Wong C.M. (2022). Peroxiredoxin, Senescence, and Cancer. Cells.

[83] Yamaguchi R., Guo X., Zheng J., Zhang J., Han J., Shioya A., Uramoto H., Mochizuki T., Yamada S. (2021). PRDX4 Improved Aging-Related Delayed Wound Healing in Mice. J. Investig. Dermatol..

[84] Gilhar A., Pillar T., David M., Eidelman S. (1991). Melanocytes and Langerhans cells in aged versus young skin before and after transplantation onto nude mice. J. Investig. Dermatol..

[85] Victorelli S., Lagnado A., Halim J., Moore W., Talbot D., Barrett K., Chapman J., Birch J., Ogrodnik M., Meves A. (2019). Senescent human melanocytes drive skin ageing via paracrine telomere dysfunction. EMBO J..

[86] Tan C.Y.R., Tan C.L., Chin T., Morenc M., Ho C.Y., Rovito H.A., Quek L.S., Soon A.L., Lim J.S.Y., Dreesen O. (2022). Nicotinamide Prevents UVB- and Oxidative Stress–Induced Photoaging in Human Primary Keratinocytes. J. Investig. Dermatol..

[87] Madreiter-Sokolowski C.T., Hiden U., Krstic J., Panzitt K., Wagner M., Enzinger C., Khalil M., Abdellatif M., Malle E., Madl T. (2024). Targeting organ-specific mitochondrial dysfunction to improve biological aging. Pharmacol. Ther..

[88] Martic I., Papaccio F., Bellei B., Cavinato M. (2023). Mitochondrial dynamics and metabolism across skin cells: Implications for skin homeostasis and aging. Front. Physiol..

[89] Shin J.W., Kwon S.H., Choi J.Y., Na J.I., Huh C.H., Choi H.R., Park K.C. (2019). Molecular Mechanisms of Dermal Aging and Antiaging Approaches. Int. J. Mol. Sci..

[90] de Vasconcelos Nasser Caetano L., de Oliveira Mendes T., Bagatin E., Amante Miot H., Marques Soares J.L., Simoes E Silva Enokihara M.M., Abrahao Martin A. (2017). In vivo confocal Raman spectroscopy for intrinsic aging and photoaging assessment. J. Dermatol. Sci..

[91] O’Reilly S., Markiewicz E., Idowu O.C. (2024). Aging, senescence, and cutaneous wound healing—A complex relationship. Front. Immunol..

[92] Thau H., Gerjol B.P., Hahn K., von Gudenberg R.W., Knoedler L., Stallcup K., Emmert M.Y., Buhl T., Wyles S.P., Tchkonia T. (2025). Senescence as a molecular target in skin aging and disease. Ageing Res. Rev..

[93] Mansfield L., Ramponi V., Gupta K., Stevenson T., Mathew A.B., Barinda A.J., Herbstein F., Morsli S. (2024). Emerging insights in senescence: Pathways from preclinical models to therapeutic innovations. NPJ Aging.

[94] Martin P., Pardo-Pastor C., Jenkins R.G., Rosenblatt J. (2024). Imperfect wound healing sets the stage for chronic diseases. Science.

[95] Gansevoort M., Oostendorp C., Bouwman L.F., Tiemessen D.M., Geutjes P.J., Feitz W.F.J., van Kuppevelt T.H., Daamen W.F. (2024). Collagen-Heparin-FGF2-VEGF Scaffolds Induce a Regenerative Gene Expression Profile in a Fetal Sheep Wound Model. Tissue Eng. Regen. Med..

[96] Libby J.R., Royce H., Walker S.R., Li L. (2024). The role of extracellular matrix in angiogenesis: Beyond adhesion and structure. Biomater. Biosyst..

[97] Chocarro-Wrona C., Pleguezuelos-Beltrán P., López de Andrés J., Antich C., de Vicente J., Jiménez G., Arias-Santiago S., Gálvez-Martín P., López-Ruiz E., Marchal J.A. (2025). A bioactive three-layered skin substitute based on ECM components effectively promotes skin wound healing and regeneration. Mater. Today Bio.

[98] Sun J., Du J., Liu X., An J., Hu Y., Wang J., Zhu F., Feng H., Cheng S., Tian H. (2024). Chondroitin sulfate-modified tragacanth gum-gelatin composite nanocapsules loaded with curcumin nanocrystals for the treatment of arthritis. J. Nanobiotechnol..

[99] Bogdanowicz P., Bensadoun P., Noizet M., Béganton B., Philippe A., Alvarez-Georges S., Doat G., Tourette A., Bessou-Touya S., Lemaitre J.M. (2024). Senomorphic activity of a combination of niacinamide and hyaluronic acid: Correlation with clinical improvement of skin aging. Sci. Rep..

[100] Jia B.B., Sun B.K., Lee E.Y., Ren B. (2024). Emerging Techniques in Spatial Multiomics: Fundamental Principles and Applications to Dermatology. J. Investig. Dermatol..

[101] Derosa G., D’Angelo A., Romano D., Maffioli P. (2017). Evaluation of the Effects of Mesoglycan on Some Markers of Endothelial Damage and Walking Distance in Diabetic Patients with Peripheral Arterial Disease. Int. J. Mol. Sci..

[102] Castanet J., Ortonne J.P. (1997). Pigmentary changes in aged and photoaged skin. Arch. Dermatol..

[103] Gilchrest B.A. (1996). A review of skin ageing and its medical therapy. Br. J. Dermatol..

[104] Liu Z.L., Chen H.H., Zheng L.L., Sun L.P., Shi L. (2023). Angiogenic signaling pathways and anti-angiogenic therapy for cancer. Signal Transduct. Target. Ther..

[105] Patel S.A., Nilsson M.B., Le X., Cascone T., Jain R.K., Heymach J.V. (2023). Molecular Mechanisms and Future Implications of VEGF/VEGFR in Cancer Therapy. Clin. Cancer Res..

[106] McMullan R.R., McAuley D.F., O’Kane C.M., Silversides J.A. (2024). Vascular leak in sepsis: Physiological basis and potential therapeutic advances. Crit. Care.

[107] Luengas-Martinez A., Ismail D., Paus R., Young H.S. (2023). Inhibition of vascular endothelial growth factor-A downregulates angiogenesis in psoriasis: A pilot study. Skin Health Dis..

[108] Ahmed I., John P., Bhatti A. (2023). Association analysis of Vascular Endothelial Growth Factor-A (VEGF-A) polymorphism in rheumatoid arthritis using computational approaches. Sci. Rep..

[109] Mangoni A.A., Zinellu A. (2024). The vascular endothelial growth factor as a candidate biomarker of systemic lupus erythematosus: A GRADE-assessed systematic review and meta-analysis. Clin. Exp. Med..

[110] Feliers D. (2009). Vascular endothelial growth factor as a prognostic marker of lupus nephritis. Kidney Int..

[111] Marneros A.G. (2016). Increased VEGF-A promotes multiple distinct aging diseases of the eye through shared pathomechanisms. EMBO Mol. Med..

[112] Chen Y., Tai Z., Zhu C., Yu Q., Zhu Q., Chen Z. (2023). Vascular Endothelial Growth Factor A (VEGFA) Inhibition: An Effective Treatment Strategy for Psoriasis. Int. J. Mol. Sci..

[113] Hartono S.P., Bedell V.M., Alam S.K., O’Gorman M., Serres M., Hall S.R., Pal K., Kudgus R.A., Mukherjee P., Seelig D.M. (2022). Vascular Endothelial Growth Factor as an Immediate-Early Activator of Ultraviolet-Induced Skin Injury. Mayo Clin Proc..

[114] Böhm M., Stegemann A., Paus R., Kleszczyński K., Maity P., Wlaschek M., Scharffetter-Kochanek K. (2025). Endocrine Controls of Skin Aging. Endocr. Rev..

[115] Wang Z., Man M.Q., Li T., Elias P.M., Mauro T.M. (2020). Aging-associated alterations in epidermal function and their clinical significance. Aging.

[116] Russell-Goldman E., Murphy G.F. (2020). The Pathobiology of Skin Aging: New Insights into an Old Dilemma. Am. J. Pathol..

[117] Zhang M., Lin Y., Han Z., Huang X., Zhou S., Wang S., Zhou Y., Han X., Chen H. (2024). Exploring mechanisms of skin aging: Insights for clinical treatment. Front. Immunol..

[118] Tufano A., Arturo C., Cimino E., Di Minno M.N., Di Capua M., Cerbone A.M., Di Minno G. (2010). Mesoglycan: Clinical evidences for use in vascular diseases. Int. J. Vasc. Med..

[119] Kitchens B.P., Snyder R.J., Cuffy C.A. (2020). A Literature Review of Pharmacological Agents to Improve Venous Leg Ulcer Healing. Wounds.

[120] Goel H.L., Mercurio A.M. (2013). VEGF targets the tumour cell. Nat. Rev. Cancer.

[121] Cialdai F., Bacci S., Zizi V., Norfini A., Balsamo M., Ciccone V., Morbidelli L., Calosi L., Risaliti C., Vanhelden L. (2022). Optimization of an Ex-Vivo Human Skin/Vein Model for Long-Term Wound Healing Studies: Ground Preparatory Activities for the ‘Suture in Space’ Experiment Onboard the International Space Station. Int. J. Mol. Sci..

[122] Gvirtz R., Ogen-Shtern N., Cohen G. (2020). Kinetic Cytokine Secretion Profile of LPS-Induced Inflammation in the Human Skin Organ Culture. Pharmaceutics.

[123] Tiirikainen M.L., Woetmann A., Norsgaard H., Santamaria-Babí L.F., Lovato P. (2020). Ex vivo culture of lesional psoriasis skin for pharmacological testing. J. Dermatol. Sci..

[124] Liu Y., Ilić T., Pantelic I., Savić S., Lunter D.J. (2022). Topically applied lipid-containing emulsions based on PEGylated emulsifiers: Formulation, characterization, and evaluation of their impact on skin properties ex vivo and in vivo. Int. J. Pharm..

[125] Oláh A., Alam M., Chéret J., Kis N.G., Hegyi Z., Szöllősi A.G., Vidali S., Bíró T., Paus R. (2020). Mitochondrial energy metabolism is negatively regulated by cannabinoid receptor 1 in intact human epidermis. Exp. Dermatol..

[126] Laufer Britva R., Keren A., Bertolini M., Ullmann Y., Paus R., Gilhar A. (2023). Involvement of ILC1-like innate lymphocytes in human autoimmunity, lessons from alopecia areata. eLife.

[127] Lu Z., Hasse S., Bodo E., Rose C., Funk W., Paus R. (2007). Towards the development of a simplified long-term organ culture method for human scalp skin and its appendages under serum-free conditions. Exp. Dermatol..

[128] Takaya K., Asou T., Kishi K. (2023). *Cistanche deserticola* Polysaccharide Reduces Inflammation and Aging Phenotypes in the Dermal Fibroblasts through the Activation of the NRF2/HO-1 Pathway. Int. J. Mol. Sci..

[129] George M., Reddy A.P., Reddy P.H., Kshirsagar S. (2024). Unraveling the NRF2 confusion: Distinguishing nuclear respiratory factor 2 from nuclear erythroid factor 2. Ageing Res. Rev..

