# Supplementary material for: Preclinical Evidence That Mesoglycan Unfolds Complex Anti-Aging Effects in Photoaged Female Facial Skin
Source: Int J Mol Sci. 2025 Jun 17;26(12):5787. doi: 10.3390/ijms26125787 (PMC12192827; doi:10.3390/ijms26125787)
Supplement: Supplementary file 1 [file ijms-26-05787-s001.zip › ijms-3609678-supplementary.pdf]

Supplementary Materials and Methods

Preparation of Full-Thickness Human Skin for Topical and Systemic Treatment

Seventy full-thickness fragments in total (5X5-mm, ten fragments from each donor) (thirty for the topical and forty for the systemic experiments) were washed in William's E medium (Gibco) supplemented with 100 IU/ml penicillin and 10 µg/ml streptomycin (Sartorius), 2 mmol/l of glutamine (Invitrogen), 10 µg/ml of insulin (Sigma) and 10 ng/ml hydrocortisone (Sigma) as described [S1,74].
For the topical experiment the skin fragments were placed in a Cell Strainer (SPL, 93040) in the medium (5.5 ml per well, 6 well plate), alternately, for the systemic experiment, skin fragments were left to float in the medium (1 ml per well, 24 well plate). For both experiments the epidermis up at air/liquid interface and the dermis/subcutis down. The cultures were maintained at 37°C in a gassed incubator with 95% air and 5% CO2. The culture medium was changed every other day.

Immunohistochemical and Immunofluorescence Staining

The human skin fragments were harvested and fixed in 10% saline-buffered formalin overnight followed by 70% ethanol. Five µm paraffin sections were either stained and visualized with hematoxylin and eosin or were taken for immunohistochemical analysis.

Antigen retrieval was performed on the slides for 20 minutes in a microwave followed by cooling at room temperature for 25 minutes. The specimens were blocked for 30 minutes to prevent nonspecific binding and incubated with the primary antibody (Ab) overnight.

The targeted markers included $p16^{INK4A}$ [48,49] and Lamin B1 [53,54], cell proliferation, Ki-67 [46,47], c-KIT [63], gp100[40] separately and gp100/MITF and c-KIT/gp100 [63], Laminin [40,41], Filaggrin [42], and CD31 [65], MTCO-1[14,38] and peroxisome proliferator-activated receptor gamma coactivator 1-alpha (PGC1α) [71],and VDAC (Porin) [14] expression. NRF-2 [71,72,S2,S3], heme Oxygenase-1 (HO-1) [79], peroxiredoxin (PRDX) [82], glutathione reductase [81], sirtuin 1 (SIRT1) levels [S2,49], p-S6 [48,52], VEGF and VEGFR2 expression [70], Masson's trichrome

[64] Picrosirius red staining [S2] and fibrillin-1 [14].Analysis using Image J software facilitated quantification of staining intensities and coverage, providing insight into the regenerative capacity of aged skin upon mesoglycan treatment.

The samples were then washed and incubated for 30 minutes with a biotinylated secondary antibody (Jackson ImmunoResearch, West Grove, PA). Finally, the samples were washed and incubated with streptavidin-horseradish peroxidase (Jackson ImmunoResearch, West Grove, PA). The proteins were revealed by treating the sections with 3-amino-9-ethylcarbazole. The samples were examined using light microscopy. Alternately, samples were incubate with 488/594 nm goat anti mouse/rabbit secondary antibodies. Nuclei were stained with DAPI.

Supplementary references

S1. Lu, Z.; Hasse, S.; Bodo, E.; Rose, C.; Funk, W.; Paus, R. Towards the development of a simplified long-term organ culture method for human scalp skin and its appendages under serum-free conditions. *Exp Dermatol*. **2007**, *16*, 37–44.

S2. Takaya, K.; Asou, T.; Kishi, K. *Cistanche deserticola* Polysaccharide Reduces Inflammation and Aging Phenotypes in the Dermal Fibroblasts through the Activation of the NRF2/HO-1 Pathway. *Int. J. Mol. Sci.* **2023**, *24*, 15704.

S3. George, M.; Reddy, A.P.; Reddy, P.H.; Kshirsagar, S. Unraveling the NRF2 confusion: Distinguishing nuclear respiratory factor 2 from nuclear erythroid factor 2. *Ageing Res. Rev.* **2024**, *98*, 102353.

Supplementary Figures

Figure S1

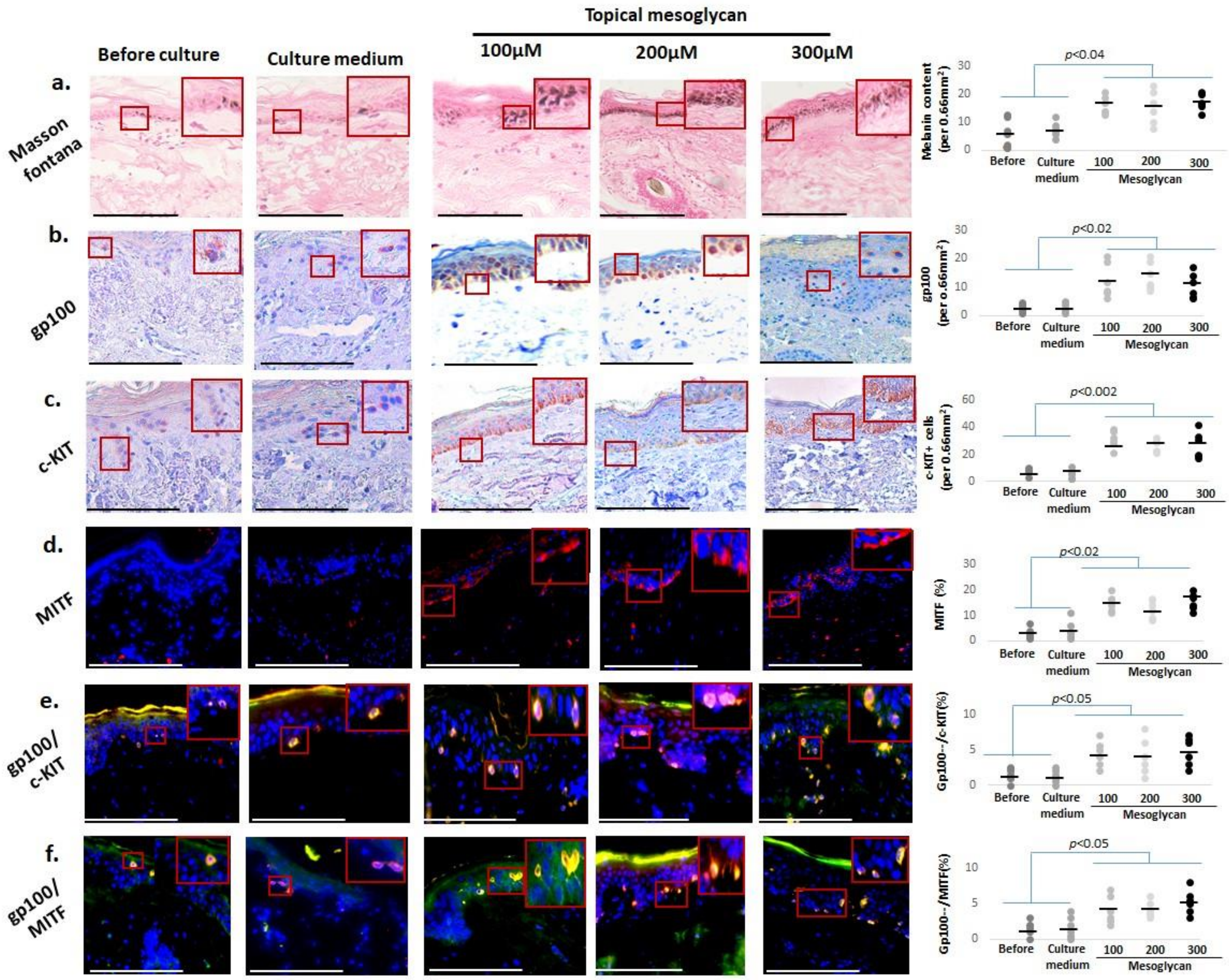

Supplementary Figure S1. Topical mesoglycan enhances pigmentary markers in aged human epidermis *ex vivo*.

Mesoglycan treatment (100–300 µM) significantly increased (**a**) melanin content (Fontana Masson staining), and expression of (**b**) gp100, (**c**) c-KIT, (**d**) MITF, and co-expression of (**e**) gp100/c-KIT and (**f**) gp100/MITF. Quantitative immunohistomorphometry and representative images illustrate these changes. Gray and black dots represent individual donors, and the horizontal bar indicates the group average. Red boxes on the micrographs mark the exact regions of interest that were quantified in the adjacent plots (see Methods 4.7 for details). Data: Mean ± SEM from three sections per group (two skin samples, three donors).Images were taken under ×200 magnification. Statistical analysis: Shapiro-Wilk, One-way ANOVA or Mann–Whitney U test ($p < 0.05$). Scale bars: 50 µm.

Figure S2

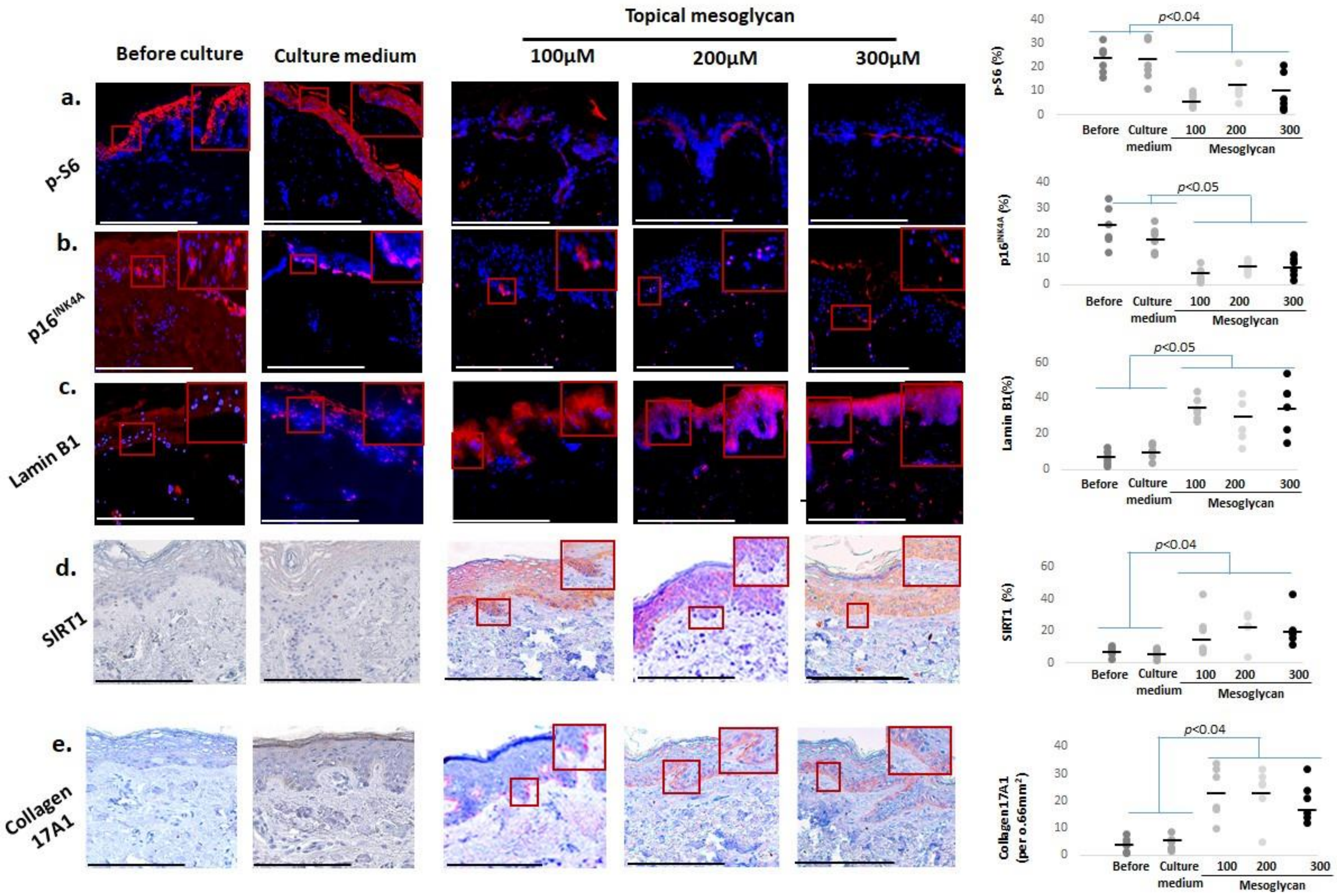

Supplementary Figure S2. Topical mesoglycan modulates senescence and rejuvenation markers in human epidermis *ex vivo*.

Mesoglycan treatment (100–300 µM) reduced expression of (**a**) p-S6 and (**b**) $p16^{INK4A}$, while increasing (**c**) Lamin B1, (**d**) SIRT1, and (**e**) Collagen 17A1 compared to baseline and medium-only controls. Quantitative immunohistomorphometry and representative images illustrate these changes. Gray and black dots represent individual donors, and the horizontal bar indicates the group average. Red boxes on the micrographs mark the exact regions of interest that were quantified in the adjacent plots (see Methods 4.7 for details). Data: Mean ± SEM from three sections per group (two skin samples, four donors). Images were taken under ×200 magnification. Statistical analysis: Shapiro-Wilk, One-way ANOVA or Mann–Whitney U test ($p < 0.05$). Scale bars: 50 µm.

Figure S3

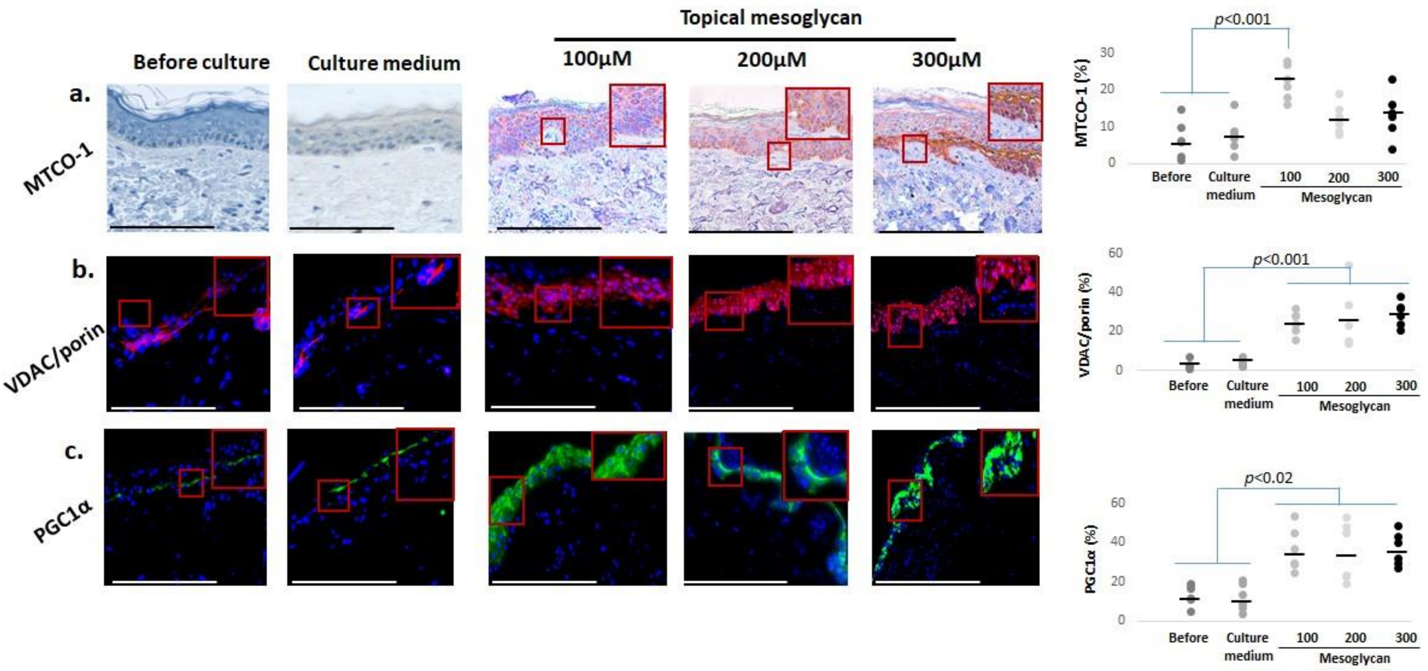

Supplementary Figure S3. Topical mesoglycan enhances mitochondrial markers in aged human epidermis *ex vivo*.

Mesoglycan treatment (100–300 µM) significantly increased expression of (**a**) MTCO-1, (**b**) VDAC/porin, and (**c**) PGC1α compared to baseline and medium-only controls. Quantitative immunohistomorphometry and representative images illustrate marker expression across treatment groups. Gray and black dots represent individual donors, and the horizontal bar indicates the group average. Red boxes on the micrographs mark the exact regions of interest that were quantified in the adjacent plots (see Methods 4.7 for details). Data: Mean ± SEM from three sections per group (two skin samples, four donors). Images were taken under ×200 magnification. Statistical analysis: Shapiro-Wilk, One-way ANOVA or Mann–Whitney U test ($p < 0.05$). Scale bars: 50 µm.

Figure S4

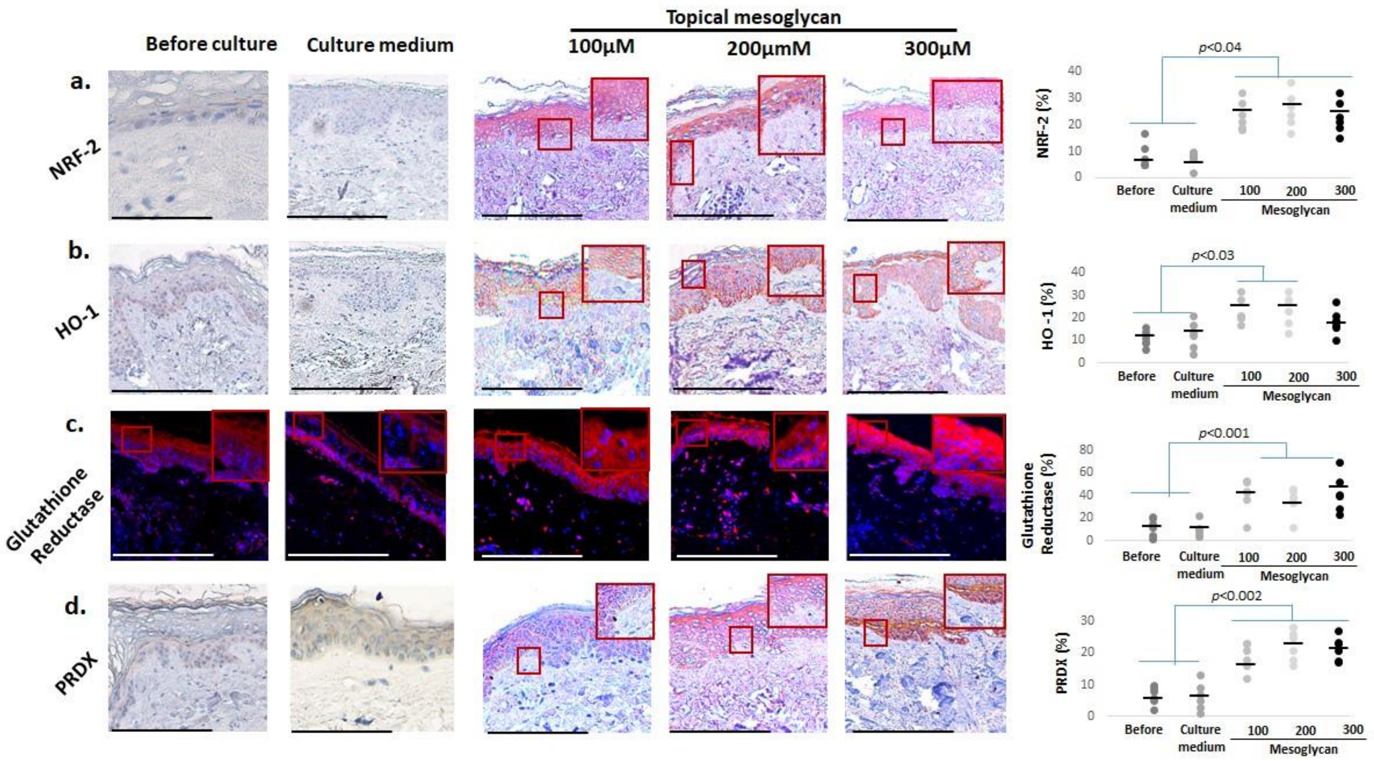

Supplementary Figure S4. Topical mesoglycan enhances NRF-2, HO-1, glutathione reductase and PRDX expression in aged human epidermis. Topical application of mesoglycan at concentrations of 100 µM, 200 µM, and 300 µM markedly increased the expression of key biomarkers: (**a**) NRF-2, (**b**) HO-1, (**c**) Glutathione Reductase and (**d**) PRDX. Quantitative immunohistomorphometry and representative images demonstrate the evaluated areas across treatment groups. Gray and black dots represent individual donors, and the horizontal bar indicates the group average. Red boxes on the micrographs mark the exact regions of interest that were quantified in the adjacent plots (see Methods 4.7 for details). Data are presented as Mean ± SEM from three non-consecutive sections from two skin samples per group, collected from three donors. Images were taken under ×200 magnification. Statistical significance was assessed using the Shapiro-Wilk test followed by One Way ANOVA or Mann-Whitney U test, with $p < 0.05$ considered significant. Scale bars: 50 µm.

Figure S5

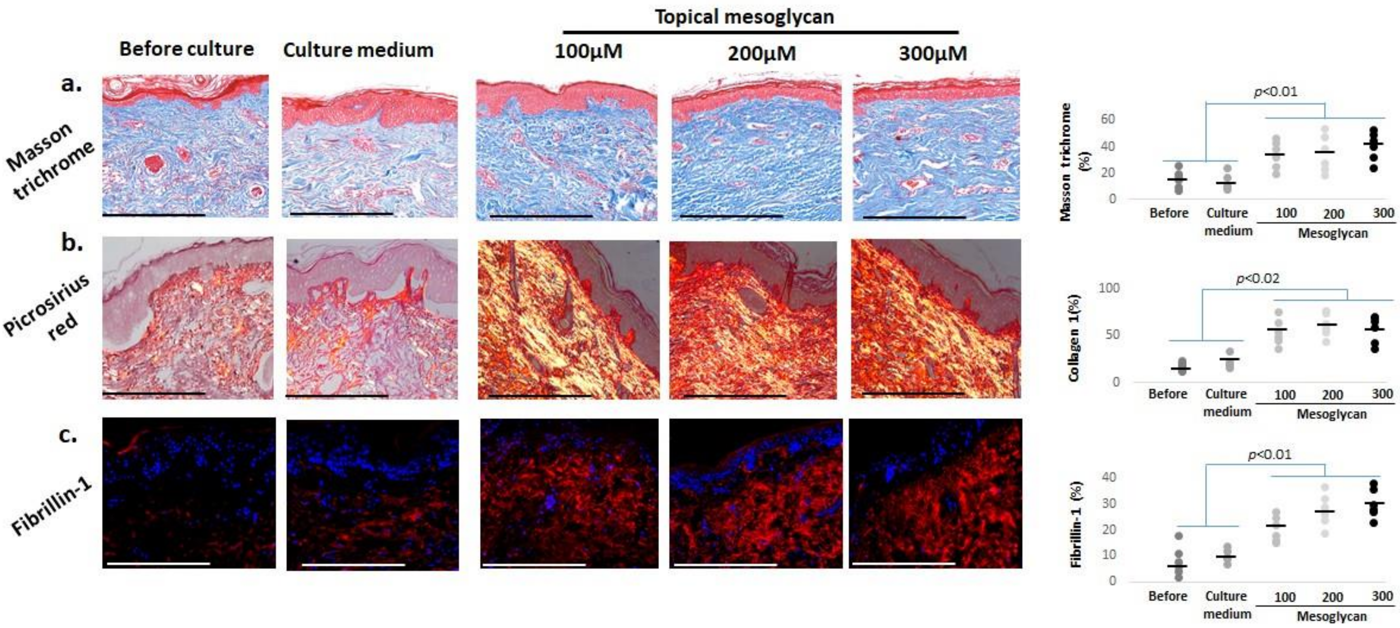

Supplementary Figure S5. Topical mesoglycan increases dermal collagen staining and fibrillin-1 expression in photoaged human skin *ex vivo*. Mesoglycan treatment (100–300 µM) significantly increased signal intensity for (**a**) Masson's trichrome, (**b**) Picrosirius red staining, and (**c**) fibrillin-1 expression compared to controls. Quantitative immunohistomorphometry and representative images illustrate these changes across treatment groups. Gray and black dots represent individual donors, and the horizontal bar indicates the group average. Red boxes on the micrographs mark the exact regions of interest that were quantified in the adjacent plots (see Methods 4.7 for details). Data are presented as Mean ± SEM from three sections per group (two skin samples, three donors). Images were taken under ×200 magnification. -Statistical analysis: Shapiro-Wilk, followed by One-way ANOVA or Mann–Whitney U test ($p < 0.05$). Scale bars: 50 µm.

Figure S6

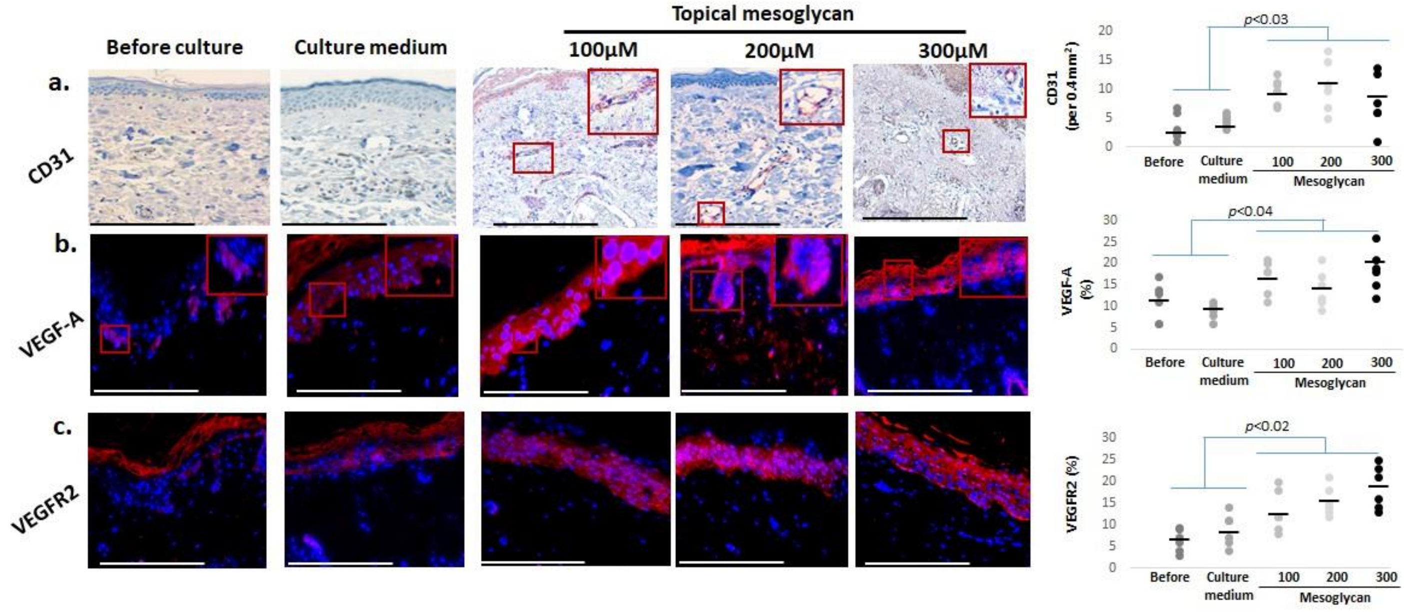

Supplementary Figure S6. Topical mesoglycan increases vascular markers in aged human skin *ex vivo*. Treatment with mesoglycan (100–300 µM) significantly elevated (**a**) CD31+ cell counts and expression of (**b**) VEGF-A and (**c**) VEGFR2. Quantitative immunohistomorphometry and representative images illustrate these changes across treatment groups. Gray and black dots represent individual donors, and the horizontal bar indicates the group average. Red boxes on the micrographs mark the exact regions of interest that were quantified in the adjacent plots (see Methods 4.7 for details). Data: Mean ± SEM from three sections per group (two skin samples, three donors). Images were taken under ×200 magnification. Statistical analysis: Shapiro-Wilk, followed by One-way ANOVA or Mann–Whitney U test ($p < 0.05$). Scale bars: 50 µm.

Supplementary Table 1: Key biomarkers and techniques for studying skin Aging mechanisms

Marker	Mechanism in aging	Ref.
Ki-67	Ki-67 is an indicator of proliferative activity, expressed during active cell cycling, but absent in quiescent (Go) cells. In aged skin, Ki-67 levels are typically reduced, reflecting a decline in the proliferative capacity of basal epidermal cells. This reduction contributes to thinning of the epidermis, and diminished ability to respond to damage, all of which are characteristic of aging skin.	[46,47]
Filaggrin	Filaggrin is an essential protein required for the proper formation and function of the skin barrier. In aged skin, filaggrin expression is often reduced, resulting in impaired skin barrier function, dryness, and increased vulnerability to environmental stressors.	[84]
Collagen XVII (COL17A1)	A key stem-cell-niche-associated transmembrane matrix molecule is expressed by epidermal stem cells, contributing to tissue homeostasis. Reduced expression of COL17A1 is closely related with epidermal aging characterized by loss of dermal-epidermal junction and epidermal thickness.	[71]
gp100	Specific to melanocyte lineage and identified by tumor-infiltrating lymphocytes, is pivotal in enabling the immune system to target melanoma cells. It is involved in melanin synthesis, playing a significant role in skin pigmentation.	[59]
MITF	**MITF (Microphthalmia-Associated Transcription Factor)** is a crucial regulator of melanocyte development, function, and survival, controlling genes involved in melanin production and pigmentation. In aged skin, MITF expression may be altered, leading to changes in pigmentation, reduced melanocyte activity, and increased susceptibility to oxidative stress.	[60,61]
c-KIT	A receptor tyrosine kinase, plays a pivotal role in the regulation of melanocyte function, including their proliferation, survival, migration, and melanin synthesis.	[63]
$P16^{INK4A}$	A protein that acts as a key regulator of cell cycle arrest, effectively halting cell division. It's often linked to aging and the onset of cellular senescence, serving as a biomarker for aged cells	[49,50]
NRF2	Nuclear factor erythroid 2-related factor 2 (NRF2) is a transcription factor best known for its crucial role in mediating the cellular antioxidant response its activity may decline with age, contributing to increased oxidative stress and reduced regenerative capacity in aged skin.	[71,72,S2,S3]
HO-1	Heme Oxygenase-1 (HO-1) is a stress-responsive enzyme that plays a role in protecting cells from oxidative damage. It catalyzes the degradation of heme into biliverdin, carbon monoxide, and free iron.	[79]
Glutathione reductase,	**Glutathione reductase** is an enzyme that helps maintain cellular redox balance by regenerating reduced glutathione (GSH) from its oxidized form (GSSG), thereby protecting cells from oxidative stress and damage.	[81]
PRDX	**Peroxiredoxins (PRDXs)** are a family of antioxidant enzymes that play a role in neutralizing hydrogen peroxide and other reactive oxygen species (ROS), thereby mitigating oxidative damage.	[82]
SIRT1	Sirtuin 1 (SIRT1) is a protein from the sirtuin family of enzymes that promotes DNA repair, metabolic regulation, and stress resistance by deacetylating target proteins. It plays a key role in maintaining cellular integrity and longevity by modulating pathways involved in oxidative stress, inflammation, and energy homeostasis.	[14,62]
PGC1α	**PGC1α (Peroxisome Proliferator-Activated Receptor Gamma Coactivator 1-Alpha)** is a key regulator of mitochondrial biogenesis and energy metabolism. In aged skin, reduced PGC1α expression is associated with mitochondrial dysfunction, decreased cellular energy production, and impaired skin regeneration. Its decline contributes to signs of aging, such as reduced skin elasticity, slower wound healing, and increased oxidative stress, making it a critical focus in studies of skin aging and potential rejuvenation therapies.	[71]
MTCO-1	MTCO-1 (Mitochondrial Cytochrome c Oxidase Subunit 1) is a key component of the cytochrome c oxidase complex (Complex IV) in the mitochondrial respiratory chain, facilitating the transfer of electrons to oxygen. In aged skin, MTCO-1 expression and activity often decline, resulting in reduced mitochondrial function,	[14]

	decreased ATP production, and increased oxidative stress, all of which contribute to impaired skin repair and accelerated aging.	
p-S6	p-S6 (Phosphorylated Ribosomal Protein S6) A sensitive direct downstream target of mTORC1 signaling, as an aging biomarker. p-S6 is associated with processes such as keratinocyte proliferation, collagen synthesis, and overall skin regeneration.	[48,52]
Lamin B1	A key component of the nuclear lamina, plays a crucial role in stabilizing the nucleus and its function tends to decline with age and cellular senescence.	[53,54]
VDAC/Porin	VDAC (Voltage-Dependent Anion Channel), also known as Porin, is a protein located in the outer mitochondrial membrane , enabling ion and metabolite exchange between mitochondria and cytoplasm. It's key in regulating apoptosis, mitophagy, and controlling mtDNA release during inflammation, highlighting its fundamental role in mitochondrial function.	[14]
Laminins	Laminins are key glycoproteins within the extracellular matrix, serving an important function in the structural support of tissues.Top of Form In the skin, where they help maintain the dermal-epidermal junction, supporting the stem cell niche and enabling proper skin repair and regeneration.	[40,41]
CD31	A cell surface glycoprotein expressed on endothelial cells, leukocytes, and platelets. It plays a crucial role in angiogenesis, inflammation, integrin activation, and cell-cell adhesion. CD31 is involved in signal transduction, mediating immune responses and contributing to the regulation of T-cell homeostasis and effector function. Its expression is also used as a marker for endothelial cells in studies of vascular tumors and angiogenesis.	[65]
Masson's trichrome staining	Histological technique widely used to differentiate between collagen, muscle tissue, and cellular components in various tissues, including the skin. In skin aging, Masson's Trichrome is used to detect reduced collagen density or disorganized collagen fibers.	[64]
Picrosirius red staining	A technique used to highlight collagen fibers in tissue samples, enhancing their birefringence under polarized light microscopy. This method allows for the differentiation of collagen types and provides insights into the organization of collagen fibers within the extracellular matrix. In aged skin, Picrosirius red staining highlights changes such as reduced collagen density, fragmentation, and disorganization, providing valuable insights into the effects of aging, photoaging, and treatments on skin structure and extracellular matrix remodeling.	[90]
Fibrillin-1	Fibrillin-1 is a key glycoprotein supporting skin elasticity by forming microfibrils within connective tissue, which is often reduced in aged skin, making it a critical marker for assessing rejuvenation effects. Fibrillin-1 reveals changes in structural integrity, with its expression by both keratinocytes and dermal fibroblasts in photoprotected and photoaged skin suggesting that these cell types actively maintain the microfibrillar network extending from the DEJ into the papillary dermis.	[14]
VEGF-A	Vascular Endothelial Growth Factor A (VEGF-A) is a potent signaling protein involved in angiogenesis and vasculogenesis, essential for new blood vessel formation and tissue repair. VEGF-A-mediated signaling is recognized as necessary and sufficient for the rejuvenation of a rapidly aging human organ, such as the skin, both at the morphological and molecular aging marker	[69,70]
VEGF-A receptor 2	The VEGF-A receptor, primarily known as VEGFR-2, is the main receptor mediating the biological effects of VEGF-A. VEGFR-2, a tyrosine kinase receptor on the surface of endothelial cells, is activated by VEGF-A binding. This activation enhances skin rejuvenation and vascularization.	[69,70]

Supplementary Table S1. Overview of biomarkers and staining techniques used to study aging mechanisms in skin. The table highlights key markers involved in cellular proliferation, skin barrier -associated proteins, mitochondrial function, oxidative stress, pigmentation, and vascularization. It also includes histological staining methods and their relevance to assessing collagen structure and elastic fiber–associated features.

Supplementary Table S2. Overview of primary antibodies and staining conditions for immunohistochemistry and immunofluorescence

Antigen (Ag)	Origin & Specificity	Brand	Cat. No.	Titer (g/ml)	Protocol used for Ag Retrieval/ Fixation
Filaggrin	Mouse anti-human	Abcam	Ab218395	1	Sodium citrate
KI-67	Rabbit anti-human	Abcam	Ab15880	1.4	Sodium citrate
gp100	Rabbit anti-human	Abcam	Ab137078	1	EDTA
c-KIT	Mouse anti-human	Invitrogen	MA5-12944	1	Sodium citrate
MITF	Rabbit anti-human	Abcam	Ab303531	1.4	EDTA
$p16^{INK4a}$	Mouse anti-human	Ventana	725-4713	1	Sodium citrate
Collagen17A	Rabbit anti-human	Abcam	EPR-14758	1	EDTA
SIRT1	Rabbit anti-human	Abcam	Ab32441	0.5	EDTA
VEGF-A	Mouse anti-human	Abcam	Ab1316	2	EDTA
VEGF-A receptor 2	Mouse anti-human	Abcam	ab315238	1	EDTA
CD31	Mouse anti-human	Invitrogen	WM-59	1.4	EDTA
MTCO-1	Rabbit anti-human	Abcam	Ab45918	1	Sodium citrate
PGC1α	Rabbit anti-human	Abcam	Ab54481	2	Sodium citrate
PRDX	Rabbit anti-human	Abcam	Ab15571	1	EDTA
HO-1	Mouse anti-human	Abcam	Ab13248	2	EDTA
p-S6	Rabbit anti-human	Invitrogen	MA5-16397	1	Sodium citrate
Lamin B1	Rabbit anti-human	Thermo Fisher Scientific	PA5-19468	1.4	Sodium citrate
Porin/VDAC	Rabbit anti-human	Thermo Fisher Scientific	55259-1-AP	0.8	EDTA
Glutathione reductase	Rat anti-human	Abcam	Ab106843	1	EDTA
Fibrillin-1	Mouse anti-human	Abcam	Ab315384	1	EDTA

Supplementary Table S2. Antibody specifications for immunohistochemistry and immunofluorescence analyses. This table summarizes each primary antibody used for tissue staining, detailing the target antigen, host species, working dilution, and supplier.
